# Supplementary material for: Implementation of medicines pricing policies in sub-Saharan Africa: protocol for a systematic review
Source: BMJ Open. 2021 Feb 23;11(2):e044293. doi: 10.1136/bmjopen-2020-044293 (PMC7907884; doi:10.1136/bmjopen-2020-044293)
Supplement: Supplementary data [file bmjopen-2020-044293supp002.pdf]

Along with Medline we intend to search the following databases:

African Index Medicus (via WHO Global Health Index Medicus) all available years; Embase (Ovid) 1996 present; Global Health (Ovid) 1973 to present; Scopus (Elsevier B.V.) 1823 – Present; Web of Science Core Collection: Citation Indexes (Clarivate Analytics) 1900-present;. We will also search for grey literature and French articles in the following; Cairn International (Cairn Info) all available years; Erudit (University of Montreal ???) all available years; IRIS Institutional Repository for Information Sharing (WHO) all available years and World Bank Group Research and Publications

Sample Medline strategy

Ovid MEDLINE(R) and Epub Ahead of Print, In-Process & Other Non-Indexed Citations and Daily <1946 to April 23, 2020>

Search Strategy:

----

1 exp "Africa South of the Sahara"/ (205870)

2 (angola* or benin* or botswana* or "burkina faso" or burundi*).ti,ab,in,kf. (17906)

3 ("cabo verde*" or "cape verde*" or cameroon* or "central africa*" or chad or cormoros or congo* or "ivory coast" or "cote d'ivoire" or djibouti).ti,ab,in,kf. (45277)

4 (guinea* or eritrea* or eswatini* or swaziland* or ethiopia* or gabon* or gambia* or ghana* or guinea*).ti,ab,in,kf. (158364)

5 (kenya* or lesotho* or liberia* or madagasca* or malawi* or mali or mauritania* or mauritius or mozambique*).ti,ab,in,kf. (55553)

6 (namibia* or niger or nigeria* or rwanda*).ti,ab,in,kf. (67659)

7 ("sao tome" or principe* or senegal* or seychelles or "sierra leone*" or somali* or "south africa*" or sudan*).ti,ab,in,kf. (132358)

8 (tanzania* or togo* or uganda* or zambia* or zaire* or zimbabw*).ti,ab,in,kf. (50798)

9 (africa* adj2 ("sub sahara*" or "south* sahara*")).ti,ab,in,kf. (23957)

10 or/1-9 [sub-saharan africa] (519682)

11 Drug Costs/ (15924)

12 ((price? or pricing) adj5 (medicine? or drug? or prescription? or pharmaceutical*)).tw,kw. (4558)

13 ((cost or costs) adj5 (medicine? or drug? or prescription? or pharmaceutical*)).tw,kw. (18930)

14 (afford* adj5 (medicine? or drug? or prescription? or pharmaceutical*)).tw,kw. (1929)

15 (reimburs* adj5 (medicine? or drug? or prescription? or pharmaceutical*)).tw,kw. (2099)

16 (generic* adj5 (medicine? or drug? or prescription? or pharmaceutical*)).tw,kw. (4521)

17 exp fees, pharmaceutical/ (2413)

18 ((purchas* or procur* or expenditure*) adj5 (medicine? or drug? or prescription? or pharmaceutical*)).tw,kw. (4913)

19 ((subsid* or tariff* or incentive* or containment or transparency) adj5 (medicine? or drug? or prescription? or pharmaceutical*)).tw,kw. (2141)

20 ((fee or fees or rebate* or payment* or spend* or saving*) adj5 (medicine? or drug? or prescription? or pharmaceutical*)).tw,kw. (4006)

21 ((benchmark* or cost-plus) adj12 (medicine? or drug? or prescription? or pharmaceutical*)).tw,kw. (641)

22 "low* price* generic*".tw,kw. (76)

23 (essential adj2 (drug* or medicine*)).tw,kw. (3434)

24 Drugs, Essential/ (835)

25 (access* adj3 (medicine? or drug? or prescription? or pharmaceutical*)).tw,kw. (5991)

26 Economics, Pharmaceutical/ (2927)

27 (pharma* adj2 economic*).tw,kw. (833)

28 pharmacoeconomic*.tw,kw. (3898)

29 or/11-28 [drug pricing] (58841)

30 exp policy/ (155119)

31 Government Regulation/ (21073)

32 exp Legislation, Drug/ (32091)

33 ((drug* or medicine* or pharmaceutical* or prescription* or health*) adj7 (guideline* or guidance or policy or policies or law or regulat* or rule* or legislat* or control* or strateg* or framework*)).tw,kw. (538082)

34 (tax or taxes or exemption*).tw,kw. (17307)

35 ((drug* or medicine* or pharmaceutical* or prescription* or health*) adj7 (intervention* or plan* or program*)).tw,kw. (256052)

36 or/30-35 [policy concept -all] (917754)

37 10 and 29 and 36 [SSA and policy] (1190)

38 Health Plan Implementation/ (5829)

39 Program Evaluation/ (62240)

40 (barrier* or facilitator* or challenge* or motivator*).tw,kw. (923252)

41 (implement* or approach* or process*).tw,kw. (3962356)

42 (factor* or determinant* or context*).tw,kw. (3829825)

43 "scal* up".tw,kw. (19228)

44 adopt*.tw,kw. (243593)

45 or/38-44 [implementation] (7698904)

46 10 and 29 and 36 and 45 (763)

47 limit 46 to yr="2000 -Current" (652)
