# Supplementary figures and images for: Implementation of medicines pricing policies in sub-Saharan Africa: protocol for a systematic review
Source: BMJ Open. 2021 Feb 23;11(2):e044293. doi: 10.1136/bmjopen-2020-044293 (PMC7907884; doi:10.1136/bmjopen-2020-044293)

**AMIPS project: Flowchart for screening of search results**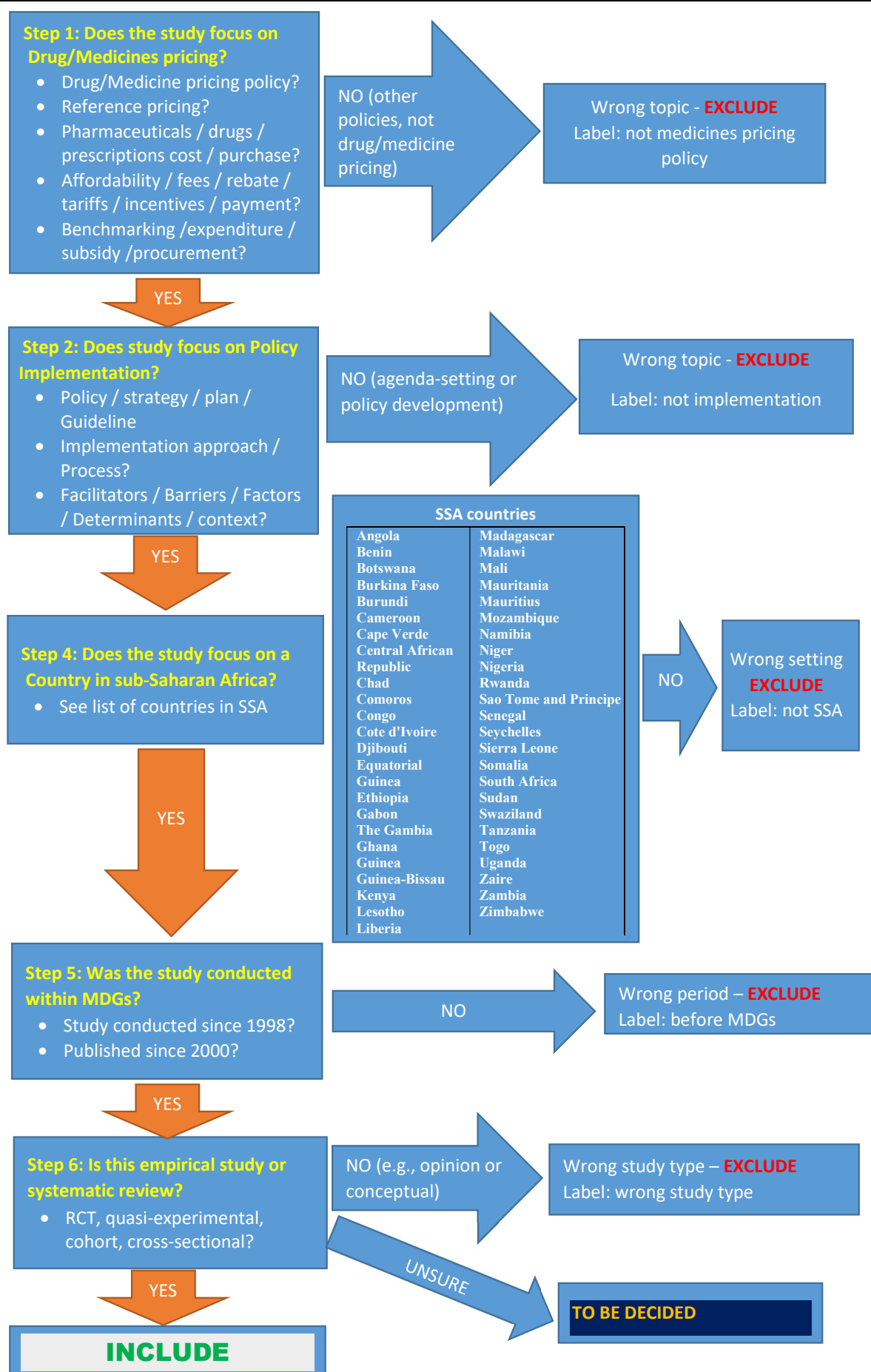

Supplement: Supplementary data [file bmjopen-2020-044293supp003.pdf]
