# Supplementary material for: Implementation of medicines pricing policies in sub-Saharan Africa: protocol for a systematic review
Source: BMJ Open. 2021 Feb 23;11(2):e044293. doi: 10.1136/bmjopen-2020-044293 (PMC7907884; doi:10.1136/bmjopen-2020-044293)
Supplement: Author's manuscript [file bmjopen-2020-044293.draft_revisions.pdf]

BMJ Open is committed to open peer review. As part of this commitment we make the peer review history of every article we publish publicly available.

When an article is published we post the peer reviewers' comments and the authors' responses online. We also post the versions of the paper that were used during peer review. These are the versions that the peer review comments apply to.

The versions of the paper that follow are the versions that were submitted during the peer review process. They are not the versions of record or the final published versions. They should not be cited or distributed as the published version of this manuscript.

BMJ Open is an open access journal and the full, final, typeset and author-corrected version of record of the manuscript is available on our site with no access controls, subscription charges or pay-per-view fees (http://bmjopen.bmj.com).

If you have any questions on BMJ Open's open peer review process please email info.bmjopen@bmj.com

Implementation of medicines pricing policies in sub-Saharan Africa: protocol for a systematic review

Journal:	*BMJ Open*
Manuscript ID	bmjopen-2020-044293
Article Type:	Protocol
Date Submitted by the Author:	29-Aug-2020

Complete List of Authors:	Mirzoev, Tolib; University of Leeds, Nuffield Centre for International Health and Development<br>Koduah, Augustina ; , School of Pharmacy, College of Health Sciences, University of Ghana, P. O. Box LG 43, Legon, Ghana<br>Cronin de Chavez, Anna; University of Leeds, Nuffield Centre for International Health and Development<br>Baatiema, Leonard; University of Ghana<br>Danso-Appiah, Anthony; University of Ghana, Regional Institute for Population Studies<br>Ensor, Tim; University of Leeds Leeds Institute of Health Sciences,<br>Agyepong, Irene; Ghana Health Service<br>Wright, Judy; University of Leeds, Leeds Institute of Health Sciences<br>Kretchy, Irene ; School of Pharmacy, University of Ghana, Department of Pharmacy Practice and Clinical Pharmacy<br>King, Natalie; University of Leeds, Leeds Institute of Health Sciences

Keywords:	Health policy < HEALTH SERVICES ADMINISTRATION & MANAGEMENT, International health services < HEALTH SERVICES ADMINISTRATION & MANAGEMENT, PUBLIC HEALTH

SCHOLARONE™ Manuscripts

BMJ

I, the Submitting Author has the right to grant and does grant on behalf of all authors of the Work (as defined in the below author licence), an exclusive licence and/or a non-exclusive licence for contributions from authors who are: i) UK Crown employees; ii) where BMJ has agreed a CC-BY licence shall apply, and/or iii) in accordance with the terms applicable for US Federal Government officers or employees acting as part of their official duties; on a worldwide, perpetual, irrevocable, royalty-free basis to BMJ Publishing Group Ltd ("BMJ") its licensees and where the relevant Journal is co-owned by BMJ to the co-owners of the Journal, to publish the Work in this journal and any other BMJ products and to exploit all rights, as set out in our licence.

The Submitting Author accepts and understands that any supply made under these terms is made by BMJ to the Submitting Author unless you are acting as an employee on behalf of your employer or a postgraduate student of an affiliated institution which is paying any applicable article publishing charge ("APC") for Open Access articles. Where the Submitting Author wishes to make the Work available on an Open Access basis (and intends to pay the relevant APC), the terms of reuse of such Open Access shall be governed by a Creative Commons licence – details of these licences and which Creative Commons licence will apply to this Work are set out in our licence referred to above.

Other than as permitted in any relevant BMJ Author's Self Archiving Policies, I confirm this Work has not been accepted for publication elsewhere, is not being considered for publication elsewhere and does not duplicate material already published. I confirm all authors consent to publication of this Work and authorise the granting of this licence.

For peer review only

Title: Implementation of medicines pricing policies in sub-Saharan Africa: protocol for a systematic review

Authors:

- Dr Tolib Mirzoev*, University of Leeds, t.mirzoev@leeds.ac.uk
- Dr Augustina Koduah, University of Ghana, akoduah@ug.edu.gh
- Dr Anna Cronin de Chavez, University of Leeds, A.Chavez@leeds.ac.uk
- Dr Leonard Baatiema, University of Ghana, baatiemaleonard@gmail.com
- Dr Anthony Danso-Appiah, University of Ghana, adanso-appiah@ug.edu.gh
- Professor Tim Ensor, University of Leeds, T.R.A.Ensor@leeds.ac.uk
- Professor Irene Agyepong, Ghana Health Service, irene.agyepong@gmail.com
- Mrs Judy Wright, University of Leeds, J.M.Wright@leeds.ac.uk
- Dr Irene Kretchy, University of Ghana, ikretchy@ug.edu.gh
- Ms Natalie King, University of Leeds, N.V.King@leeds.ac.uk

*Corresponding author: Nuffield Centre for International Health and Development, Level 10 Worsley Building, Clarendon Way, Leeds LS2 9NL. t.mirzoev@leeds.ac.uk +44(0)113-343-4869

Keywords

Systematic review; medicine pricing; policy implementation; sub-Saharan Africa; global health

Word count

2,228 words

For peer review only

Abstract

Introduction

Ensuring universal availability and accessibility of medicines and supplies is critical for national health systems to equitably address population health needs. In sub-Saharan Africa (SSA), this is a recognised policy priority with multiple medicines pricing policies enacted. However, medicine prices have remained high and continued to rise. In this systematic review, we aim to identify and analyse experiences of successful implementation of medicines pricing policies in SSA in order to inform improved implementation of Ghanaian policies.

Methods and analysis

Eight electronic databases, grey literature and reference lists from previous similar reviews will be searched. Published peer-reviewed studies of implementation of medicines pricing policies in sub-Saharan Africa will be eligible for inclusion. Broader policy analyses and documented experiences of implementation of other health policies will be excluded. The team will collaboratively screen titles and abstracts, then two reviewers will independently screen full-texts, extract data and assess quality of the included studies. Disagreements will be resolved by discussion or a third reviewer. Data will be extracted on approaches used for policy implementation, actors involved, evidence used in decision-making and key contextual influences on policy implementation. A narrative approach will be used to synthesise the data. Reporting will be informed by the Preferred Reporting Items for Systematic Review and Meta-Analysis Protocols (PRISMA) guideline.

Ethics and dissemination

No ethics approvals are required for systematic reviews.

Results will be disseminated through academic publications, policy briefs and presentations to national policymakers in Ghana.

Registration details

Prospero registration number: CRD42020178166, full record: https://www.crd.york.ac.uk/prospero/display_record.php?ID=CRD42020178166

For peer review only

Article summary

Strengths and limitations of this study

- This systematic review protocol follows the Preferred Reporting Items for Systematic Review and Meta-Analysis Protocols (PRISMA-P) guidelines
- The review addresses a gap in the current knowledge of the determinants of successful implementation of medicines pricing policies in sub-Saharan Africa
- The focus on sub-Saharan Africa (SSA) will help with transferability of lessons across the different countries within the region, though may lead to omission of important experiences for example from Asia and Latin America and may limit transferability of lessons outside the SSA.
- The search will be restricted to peer-reviewed published articles and grey literature, thus relevant theses and conference abstracts are likely to be omitted and may affect the depth of evidence on the topic.
- The narrative synthesis approach reflects the nature of published evidence on the topic of policy implementation with no meta-analysis possible, and is a potential limitation of this review

For peer review only

Introduction

The current agenda of Universal Health Coverage (UHC) highlights the importance of access to safe, quality and affordable medicines as a key driver in the attainment of the UHC.[1-3] Increasing access to essential medicines through medicines pricing interventions is also an issue of current health policy discourses. [4 5] In response, various policy initiatives have evolved to regulate medicine pricing and improve access.

Globally, different medicine pricing models and strategies exist and these include generic or biosimilar price linked to originator product, non-proprietary prescribing and generic substitution, tendering and pooled procurement, internal reference pricing, external price referencing or international price comparisons, and managed-entry agreement. [6-8] Implementation of these medicine pricing policies may be dependent on pricing levels of the medicines, whether it is generic, be it out-patient or in-patient service.[9]

Many of these medicine pricing policies are being implemented in high income countries. However, unlike high income countries, low and middle income countries (LMICs) have less regulated and developed pharmaceutical markets. [1] In light of this, multiple medicine pricing models and strategies are required to achieve equitable access to safe, quality and affordable medicines. [10], particularly, in sub-Saharan African countries (SSA). [10]

Rationale

Ensuring availability and accessibility of medicines is an important mechanism by which national health systems can equitably address the health needs of the population, including the poorest and most vulnerable. In SSA, this is a recognised policy priority. For example, in the last two decades different medicines pricing policy were implemented in South Africa [11 12] and between 2012 and 2017, the Government of Ghana introduced four policies to improve access to medicines through medicine price regulation, and ultimately health outcomes and quality of life. These policies currently at different stages of their implementation and despite these efforts, medicine prices have remained high and continued to rise in many countries. This raises questions as to why and how these policies are failing to achieve the desired outcomes.

In this review, we will explore the effectiveness of implementation of medicines pricing policies in the sub-Saharan African context. We want to identify which policies have been implemented and then explore three broad dimensions of their implementation. First, we want to understand *what happened* i.e. identify evidence on effective implementation of medicines pricing policies reflected in reduction in prices and improvement in access to medicines and subsequently healthcare. Second, we want to understand *how it happened* i.e. we will identify and unpack the implementation processes and approaches deployed in terms of their timing, participation of actors and role of evidence. Third, we want to understand *why it happened*. We will synthesise the key reported facilitators and barriers to the implementation and understand how they affected the implementation of these policies within their respective contexts.

Aim and objectives

For peer review only

In this review we will address the following overall question: what are the key determinants of successful implementation of medicines pricing policies in sub-Saharan African (SSA) countries? More specifically, we will answer four questions:

a. Which medicines pricing policies have been implemented in SSA and what are their key elements?
b. How have these policies been implemented (in relation to implementation approaches, processes, involvement of actors, role of evidence etc)?
c. Which key facilitators and barriers affected the implementation of medicines pricing policies, and how?
d. Which implementation of medicines pricing policies in SSA are effective (in relation to reducing prices of medicines and improving access to services)?

This review is being undertaken during April – November 2020 as part of the project on 'Improving equitable access to essential medicines in Ghana through bridging the gaps in implementing medicines pricing policy' (AMIPS) – a NIHR-funded award received jointly by the University of Leeds and University of Ghana. The results of this review will be combined with results of policy analyses in Ghana and will inform engagements with key stakeholders on improving the implementation of the current policies and identification of future research and development priorities on the topic.

This protocol follows the Preferred reporting items for systematic review and meta-analysis protocols (PRISMA-P) guidelines[13] and a PRISMA-P checklist is available as a supplementary file.

Methods and analysis

Eligibility criteria

Studies

We will include: both empirical studies (including Randomised Controlled Trials (RCTs), quasi-experimental studies and cohort and cross-sectional studies). Reviews (scoping reviews, meta-syntheses, realist syntheses) will be included and individual primary studies from the systematic reviews will be manually included as empirical literature. We will exclude opinion pieces and conceptual/theoretical publications, which do not report documented empirical data from either primary studies or reviews.

Specific inclusion criteria will be: (a) Focus on the medicines pricing policies i.e. policies, strategies, interventions or plans which aim to improve affordability of medicines in the country. The link to improvements in access to healthcare may be implicit and is not a requirement; (b) Focus on policy implementation i.e. either as part of the whole policy process (agenda-setting, development, implementation) or as an exclusive focus; (c) sub-Saharan African country contexts i.e. either as part of the comparative studies or as a sole focus; (d) studies which were published since the agenda of Millennium Development Goals was initiated shortly before 2000; and (e) papers with relevant information available for analysis.

Specific exclusion criteria are: (a) policy analyses which focus solely on policy agenda-setting and development stages of the policy process; (b) Studies from high-income country contexts and outside sub-Saharan Africa; (c) studies conducted two years or more prior to 2000 but published after 2000, will be excluded, in consideration of MDG and SDG agenda which started in 2000; (d) papers in languages where we are unable to have the resources for translation (the team has access to French, Spanish and Russian-speaking researchers) and (e) papers with no full text available for analysis.

For peer review only

Participants

The participants to be covered in the review will be: policymakers, implementers, service providers, patients and beneficiaries of successful implementation of medicines pricing policies (of any gender, age, ethnicity, socio-economic group, health status or urban-rural residence).

Interventions

Implementation of medicines pricing policies i.e. policies, strategies, interventions or plans which aim to improve affordability of medicines in the country.

Comparison

No comparison or control is applicable to this study

Outcomes

Successful implementation will be measured as reduction in medicines prices, and improved access to medicines along the supply chain. Any studies describing unsuccessful implementation will also be used to inform the lessons learned.

Study records

Searches

We will search relevant databases: Medline (1946 – present), Web of Science (1990 – present), Scopus (1823 – present), Global Health (1973 – present), Embase (1947 – present), Cairn.Info International Edition (all available years), Erudit (all available years) and African Index Medicus (all available years). The Medline search strategy is available as a supplementary file. The search strategies will incorporate index terms (MeSH) and text words for the search concepts:

1. Sub-Saharan African Countries. This will include terms/synonyms for sub-Saharan Africa AND list of individual countries in the region
2. Drug/Medicines pricing. This will include terms/synonyms for: medicines / pharmaceuticals / drugs / prescriptions AND pricing / cost / affordability / fees / purchase / rebate(s) / tariffs / incentives / benchmarking / reference pricing / payment / spend* / expenditure / subsid* / procurement
3. Policy. This will include terms/synonyms for: policy / strategy / plan / framework / regulations / guidelines / rules / intervention / tax / exemption
4. Implementation. This will include terms/synonyms for: Implementing / Implement(s) / Implementation approach(es) / Process(es) / Facilitator(s) / Barrier(s) / Factors / Determinants / context

The searches will be limited to the literature published from the year 2000 and onwards. This is in consideration of the Millennium Development Goals agenda which started in 2000 with a clear focus on improving access to medicines and services. We will follow-up on the references to the individual studies as required. We will manually search for the included references in relevant retrieved reviews (systematic reviews, scoping reviews, meta-syntheses, realist syntheses) for additional relevant studies for inclusion. In addition, we will search grey literature including global development websites: WHO IRIS, World Bank, DFID/K4D repository, Gates Foundation and contacts with experts in the field.

For peer review only

Data management

We will upload all references identified through searches (electronic database and additional searches) into Endnote version X9. Once duplicates are removed, the remaining references will be exported into Rayyan (https://rayyan.qcri.org/welcome), an online free systematic review tool for screening

Screening

Titles and abstracts will be divided up across the review team and screened individually for eligibility using pre-specified eligibility criteria flowchart, which is available in a supplementary file. At least 20% of individually reviewed titles and abstracts will then be cross-checked by at least two of the team.. Full texts will be obtained for all the potentially relevant studies and screened by two members of the team independently, and disagreements will be resolved through discussion. Where necessary, a third member of the team will engage to help resolve disagreements.

Data extraction

The following data will be extracted by two members of the review team into an appropriate data extraction form: article information (full citation, year study was conducted, study type, setting / country); medicine pricing policies studied (including which key elements the policies included); documented effects on prices of medicines (including how identified and reported); effects on access to medicines (including how identified and reported); effects on access to healthcare (including how identified and reported); implementation approach (including processes, actors involved and their roles and evidence used to inform implementation); key influences on policy implementation (including facilitators and constraints and how they affected implementation).

Quality assessment and risk of bias

Quality of each included study will be appraised. We will utilise validated quality assessment tools and the critical appraisal tools for relevant studies (qualitative and quantitative research) from the Joanna Briggs Institute https://joannabriggs.org/ebp/critical_appraisal_tools. While at this point, we do not intend to change the actual criteria, the interpretation and application of the tools will be within the context of our study which focuses on key determinants of effective implementation of medicines pricing policies in sub-Saharan African contexts. For example, clarity of focus will be assessed in relation to how the different aspects of policy implementation (processes, use of evidence, involvement of actors) are identified and consistently used in the reviewed papers.

A careful assessment of risk of bas in the included studies will be performed by two reviewers, who will first independently assess the quality of each study against each criterion. Results will be shared and agreed, and any disagreements will be addressed through engaging a third reviewer.

Data synthesis and interpretation

Strategy for data synthesis.

The main outcome in our study is the medicine pricing policy implementation. Policy implementation is typically done within a single country, but where the same policy is implemented in different countries, the analysis will take the specific context of the country into consideration.

In exploring the policy implementation, we will employ established policy theories and frameworks such as Walt and Gilson's policy triangle [14], Baumgartner's punctuated equilibrium [15] and Lipsky's

For peer review only - http://bmjopen.bmj.com/site/about/guidelines.xhtml

street-level bureaucracy [16], and will also draw upon further theories and frameworks developed or adapted within the reviewed papers.

Where possible, we will attempt to compare the effects of the policies in a quantitative synthesis. We anticipate, however, that the heterogeneity of reporting of outcomes and of context, may make it impossible to conduct a meta-analysis. In such a situation we will focus on narrative synthesis.

Whilst using qualitative or narrative synthesis approach [17], data related to the medicines pricing policies will be extracted from the Introduction, Methods and possibly Results. Data on the effects of policy implementation and the policy implementation approaches, and key influences will be extracted from the Results and Discussion sections. Extracted data will be analysed thematically and will be structured around the specific questions of the review.

The interpretation of the results will follow the identified themes for each review question. For example, in answering the third review question we will divide the factors into facilitators and constraints and potentially will further sub-divide them by their nature (e.g. community issues, health systems issues, wider socio-economic influences).

At the moment, we are not planning analysis of sub-groups or sub-sets. However, depending on the breadth of extracted data we may consider sub-groups such as geographical region (West Africa, East Africa, Southern Africa), setting (urban, rural) or categories of implementers (health facilities, pharmacies).

Patient and public involvement

This review will use published data, therefore, no direct patient and public involvement in the design, interpretation or dissemination of the findings is planned.

Ethics and dissemination

Ethics approvals are not required for systematic reviews. However, ethics approvals for the wider AMIPS study within which this review is being undertaken have been granted by the ethics committees from the Ghana Health Service (ref GHS-ERC006/02/20) and the University of Leeds School of Medicine (ref MREC 19-060).

We shall disseminate results through academic papers and stakeholder workshops in Ghana and other SSA countries, where necessary. In Ghana, the review results will be complemented by reviews of policy documents. The findings of the review will also be presented at scientific conferences such as the Global Symposia on Health Systems Research and Thematic Working Groups of the Health Systems Global.

The results of this review will inform empirical investigations of implementation of medicines pricing policies in Ghana through the in-depth interviews and focus groups, as well as engagements and consultations with policymakers on seeking ways of further improving the implementation of medicines pricing policies in Ghana.

References

1. Bigdeli M, Laing R, Tomson G. Medicines and universal health coverage: challenges and opportunities: BioMed Central, 2015.

For peer review only

2. Ghebreyesus TA. All roads lead to universal health coverage. *The Lancet Global Health* 2017;5(9):e839-e40.
3. Wirtz VJ, Hogerzeil HV, Gray AL, et al. Essential medicines for universal health coverage. *The Lancet* 2017;389(10067):403-76.
4. World Health O, Regional Office for E. Access to new medicines in Europe : technical review of policy initiatives and opportunities for collaboration and research. 2015
5. World Health Organization. Assessment of medicine pricing and reimbursement systems in health insurance schemes. 2016
6. Bashaar M, Hassali F, ALrasheedy A, et al. Efficacy of international approaches to medicine price regulation and control: A scoping review. *J Appl Pharm Sci* 2017;7(04):227-41.
7. Leopold C, Vogler S, Mantel-Teeuwisse AK, et al. Differences in external price referencing in Europe—a descriptive overview. *Health Policy* 2012;104(1):50-60.
8. Vogler S, Paris V, Ferrario A, et al. How can pricing and reimbursement policies improve affordable access to medicines? Lessons learned from European countries. *Applied health economics and health policy* 2017;15(3):307-21.
9. Vogler S, Zimmermann N, de Joncheere K. Policy interventions related to medicines: Survey of measures taken in European countries during 2010–2015. *Health Policy* 2016;120(12):1363-77.
10. Nguyen TA, Knight R, Roughead EE, et al. Policy options for pharmaceutical pricing and purchasing: issues for low-and middle-income countries. *Health policy and planning* 2015;30(2):267-80.
11. Moodley R, Suleman F. Evaluating the impact of the single exit price policy on a basket of originator medicines in South Africa from 1999 to 2014 using a time series analysis. *BMC Health Services Research* 2019;19(1):576. doi: 10.1186/s12913-019-4403-8
12. Steyn R, Burger JR, Serfontein JHP, et al. Influence of a new reference-based pricing system in South Africa on the prevalence and cost of antidiabetic medicine: a pilot study. *International Journal of Pharmacy Practice* 2007;15(4):307-11. doi: 10.1211/ijpp.15.4.0009
13. Shamseer L, Moher D, Clarke M, et al. Preferred reporting items for systematic review and meta-analysis protocols (PRISMA-P) 2015: elaboration and explanation. *BMJ : British Medical Journal* 2015;349:g7647. doi: 10.1136/bmj.g7647
14. Walt G, Gilson L. Reforming the health sector in developing countries: the central role of policy analysis. *Health Policy Plan* 1994;9(4):353-70.
15. Baumgartner F, Jones B. Agendas and Instability in American Politics. Chicago, London: University of Chicago Press 1993.
16. Lipsky M. Street-level bureaucracy: dilemmas of the individual in public services New York: Russel Sage Foundation 1980.
17. Snilstveit B, Oliver S, Vojtkova M. Narrative approaches to systematic review and synthesis of evidence for international development policy and practice. *Journal of Development Effectiveness* 2012;4(3):409-29. doi: 10.1080/19439342.2012.710641

Authors' contributions

AK and TM jointly conceived the study; TM, AK, AC, LB, TDA, TE, IA, JW, IK, NK contributed to the review design and jointly wrote the protocol; TM, AK, AC, LB, TDA, TE, IA, JW, IK, NK read and approved the final version of the manuscript.

For peer review only

Funding

This research was commissioned by the National Institute for Health Research (NIHR) NIHR Global Health Policy and Systems Research Development Award using UK aid from the UK Government (grant number 130219). The views expressed in this publication are those of the author(s) and not necessarily those of the NIHR or the Department of Health and Social Care

Competing interests

None declared

For peer review only

Title: Implementation of medicines pricing policies in sub-Saharan Africa: protocol for a systematic review

PRISMA-P (preferred reporting items for systematic review and meta-analysis protocols) 2015 checklist: recommended items to address in a systematic review protocol

Section and topic	*Item No*	*Checklist item*	*Y/N*
**Administrative information**			
Title:			
Identification	1a	Identify the report as a protocol of a systematic review	Y
Update	1b	If the protocol is for an update of a previous systematic review, identify as such	Y
Registration	2	If registered, provide the name of the registry (such as PROSPERO) and registration number	Y
Authors:			
Contact	3a	Provide name, institutional affiliation, e-mail address of all protocol authors; provide physical mailing address of corresponding author	Y
Contributions	3b	Describe contributions of protocol authors and identify the guarantor of the review	Y
Amendments	4	If the protocol represents an amendment of a previously completed or published protocol, identify as such and list changes; otherwise, state plan for documenting important protocol amendments	n/a
Support:			
Sources	5a	Indicate sources of financial or other support for the review	Y
Sponsor	5b	Provide name for the review funder and/or sponsor	Y
Role of sponsor or funder	5c	Describe roles of funder(s), sponsor(s), and/or institution(s), if any, in developing the protocol	Y
**Introduction**			
Rationale	6	Describe the rationale for the review in the context of what is already known	Y
Objectives	7	Provide an explicit statement of the question(s) the review will address with reference to participants, interventions, comparators, and outcomes (PICO)	Y
**Methods**			
Eligibility criteria	8	Specify the study characteristics (such as PICO, study design, setting, time frame) and report characteristics (such as years considered, language, publication status) to be used as criteria for eligibility for the review	Y
Information sources	9	Describe all intended information sources (such as electronic databases, contact with study authors, trial registers or other grey literature sources) with planned dates of coverage	Y
Search strategy	10	Present draft of search strategy to be used for at least one electronic database, including planned limits, such that it could be repeated	Y
Study records:			

For peer review only

Section and topic	*Item No*	*Checklist item*	*Y/N*
Data management	11a	Describe the mechanism(s) that will be used to manage records and data throughout the review	Y
Selection process	11b	State the process that will be used for selecting studies (such as two independent reviewers) through each phase of the review (that is, screening, eligibility and inclusion in meta-analysis)	Y
Data collection process	11c	Describe planned method of extracting data from reports (such as piloting forms, done independently, in duplicate), any processes for obtaining and confirming data from investigators	Y
Data items	12	List and define all variables for which data will be sought (such as PICO items, funding sources), any pre-planned data assumptions and simplifications	Y
Outcomes and prioritization	13	List and define all outcomes for which data will be sought, including prioritization of main and additional outcomes, with rationale	Y
Risk of bias in individual studies	14	Describe anticipated methods for assessing risk of bias of individual studies, including whether this will be done at the outcome or study level, or both; state how this information will be used in data synthesis	Y
Data synthesis	15a	Describe criteria under which study data will be quantitatively synthesised	Y
	15b	If data are appropriate for quantitative synthesis, describe planned summary measures, methods of handling data and methods of combining data from studies, including any planned exploration of consistency (such as I2, Kendall's τ)	n/a
	15c	Describe any proposed additional analyses (such as sensitivity or subgroup analyses, meta-regression)	n/a
	15d	If quantitative synthesis is not appropriate, describe the type of summary planned	Y
Meta-bias(es)	16	Specify any planned assessment of meta-bias(es) (such as publication bias across studies, selective reporting within studies)	Y
Confidence in cumulative evidence	17	Describe how the strength of the body of evidence will be assessed (such as GRADE)	Y

For peer review only

Along with Medline we intend to search the following databases:

African Index Medicus (via WHO Global Health Index Medicus) all available years; Embase (Ovid) 1996 present; Global Health (Ovid) 1973 to present; Scopus (Elsevier B.V.) 1823 – Present; Web of Science Core Collection: Citation Indexes (Clarivate Analytics) 1900-present;. We will also search for grey literature and French articles in the following; Cairn International (Cairn Info) all available years; Erudit (University of Montreal ???) all available years; IRIS Institutional Repository for Information Sharing (WHO) all available years and World Bank Group Research and Publications

Sample Medline strategy

Ovid MEDLINE(R) and Epub Ahead of Print, In-Process & Other Non-Indexed Citations and Daily <1946 to April 23, 2020>

Search Strategy:

----

1 exp "Africa South of the Sahara"/ (205870)

2 (angola* or benin* or botswana* or "burkina faso" or burundi*).ti,ab,in,kf. (17906)

3 ("cabo verde*" or "cape verde*" or cameroon* or "central africa*" or chad or cormoros or congo* or "ivory coast" or "cote d'ivoire" or djibouti).ti,ab,in,kf. (45277)

4 (guinea* or eritrea* or eswatini* or swaziland* or ethiopia* or gabon* or gambia* or ghana* or guinea*).ti,ab,in,kf. (158364)

5 (kenya* or lesotho* or liberia* or madagasca* or malawi* or mali or mauritania* or mauritius or mozambique*).ti,ab,in,kf. (55553)

6 (namibia* or niger or nigeria* or rwanda*).ti,ab,in,kf. (67659)

7 ("sao tome" or principe* or senegal* or seychelles or "sierra leone*" or somali* or "south africa*" or sudan*).ti,ab,in,kf. (132358)

8 (tanzania* or togo* or uganda* or zambia* or zaire* or zimbabw*).ti,ab,in,kf. (50798)

9 (africa* adj2 ("sub sahara*" or "south* sahara*")).ti,ab,in,kf. (23957)

10 or/1-9 [sub-saharan africa] (519682)

11 Drug Costs/ (15924)

12 ((price? or pricing) adj5 (medicine? or drug? or prescription? or pharmaceutical*)).tw,kw. (4558)

13 ((cost or costs) adj5 (medicine? or drug? or prescription? or pharmaceutical*)).tw,kw. (18930)

14 (afford* adj5 (medicine? or drug? or prescription? or pharmaceutical*)).tw,kw. (1929)

15 (reimburs* adj5 (medicine? or drug? or prescription? or pharmaceutical*)).tw,kw. (2099)

16 (generic* adj5 (medicine? or drug? or prescription? or pharmaceutical*)).tw,kw. (4521)

17 exp fees, pharmaceutical/ (2413)

For peer review only

18 ((purchas* or procur* or expenditure*) adj5 (medicine? or drug? or prescription? or pharmaceutical*)).tw,kw. (4913)

19 ((subsid* or tariff* or incentive* or containment or transparency) adj5 (medicine? or drug? or prescription? or pharmaceutical*)).tw,kw. (2141)

20 ((fee or fees or rebate* or payment* or spend* or saving*) adj5 (medicine? or drug? or prescription? or pharmaceutical*)).tw,kw. (4006)

21 ((benchmark* or cost-plus) adj12 (medicine? or drug? or prescription? or pharmaceutical*)).tw,kw. (641)

22 "low* price* generic*".tw,kw. (76)

23 (essential adj2 (drug* or medicine*)).tw,kw. (3434)

24 Drugs, Essential/ (835)

25 (access* adj3 (medicine? or drug? or prescription? or pharmaceutical*)).tw,kw. (5991)

26 Economics, Pharmaceutical/ (2927)

27 (pharma* adj2 economic*).tw,kw. (833)

28 pharmacoeconomic*.tw,kw. (3898)

29 or/11-28 [drug pricing] (58841)

30 exp policy/ (155119)

31 Government Regulation/ (21073)

32 exp Legislation, Drug/ (32091)

33 ((drug* or medicine* or pharmaceutical* or prescription* or health*) adj7 (guideline* or guidance or policy or policies or law or regulat* or rule* or legislat* or control* or strateg* or framework*)).tw,kw. (538082)

34 (tax or taxes or exemption*).tw,kw. (17307)

35 ((drug* or medicine* or pharmaceutical* or prescription* or health*) adj7 (intervention* or plan* or program*)).tw,kw. (256052)

36 or/30-35 [policy concept -all] (917754)

37 10 and 29 and 36 [SSA and policy] (1190)

38 Health Plan Implementation/ (5829)

39 Program Evaluation/ (62240)

40 (barrier* or facilitator* or challenge* or motivator*).tw,kw. (923252)

41 (implement* or approach* or process*).tw,kw. (3962356)

42 (factor* or determinant* or context*).tw,kw. (3829825)

43 "scal* up".tw,kw. (19228)

For peer review only

44 adopt*.tw,kw. (243593)

45 or/38-44 [implementation] (7698904)

46 10 and 29 and 36 and 45 (763)

47 limit 46 to yr="2000 -Current" (652)

For peer review only

AMIPS project: Flowchart for screening of search results

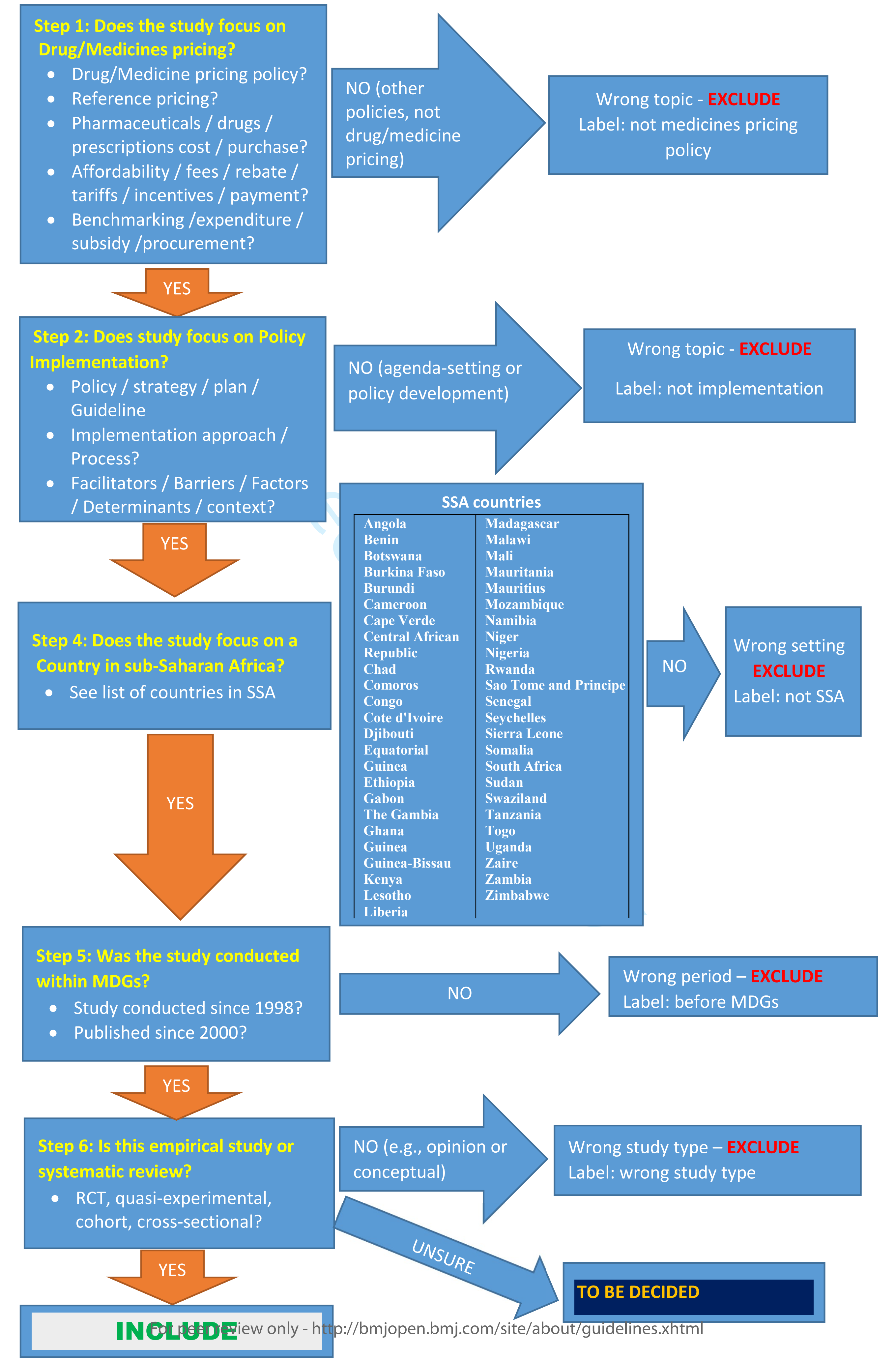

BMJ Open

Implementation of medicines pricing policies in sub-Saharan Africa: protocol for a systematic review

Journal:	*BMJ Open*
Manuscript ID	bmjopen-2020-044293.R1
Article Type:	Protocol
Date Submitted by the Author:	12-Dec-2020
Complete List of Authors:	Mirzoev, Tolib; University of Leeds, Nuffield Centre for International Health and Development<br>Koduah, Augustina ; University of Ghana, School of Pharmacy<br>Cronin de Chavez, Anna; University of Leeds, Nuffield Centre for International Health and Development<br>Baatiema, Leonard; University of Ghana, School of Pharmacy<br>Danso-Appiah, Anthony; University of Ghana, School of Public Health<br>Ensor, Tim; University of Leeds, Nuffield Centre for International Health and Development<br>Agyepong, Irene; Ghana Health Service, Research and Development Division<br>Wright, Judy; University of Leeds, Leeds Institute of Health Sciences<br>Kretchy, Irene ; University of Ghana, School of Pharmacy<br>King, Natalie; University of Leeds, Leeds Institute of Health Sciences
**Primary Subject Heading**:	Health policy
Secondary Subject Heading:	Global health
Keywords:	Health policy < HEALTH SERVICES ADMINISTRATION & MANAGEMENT, International health services < HEALTH SERVICES ADMINISTRATION & MANAGEMENT, PUBLIC HEALTH

SCHOLARONE™
Manuscripts

For peer review only - http://bmjopen.bmj.com/site/about/guidelines.xhtml

BMJ

I, the Submitting Author has the right to grant and does grant on behalf of all authors of the Work (as defined in the below author licence), an exclusive licence and/or a non-exclusive licence for contributions from authors who are: i) UK Crown employees; ii) where BMJ has agreed a CC-BY licence shall apply, and/or iii) in accordance with the terms applicable for US Federal Government officers or employees acting as part of their official duties; on a worldwide, perpetual, irrevocable, royalty-free basis to BMJ Publishing Group Ltd ("BMJ") its licensees and where the relevant Journal is co-owned by BMJ to the co-owners of the Journal, to publish the Work in this journal and any other BMJ products and to exploit all rights, as set out in our licence.

The Submitting Author accepts and understands that any supply made under these terms is made by BMJ to the Submitting Author unless you are acting as an employee on behalf of your employer or a postgraduate student of an affiliated institution which is paying any applicable article publishing charge ("APC") for Open Access articles. Where the Submitting Author wishes to make the Work available on an Open Access basis (and intends to pay the relevant APC), the terms of reuse of such Open Access shall be governed by a Creative Commons licence – details of these licences and which Creative Commons licence will apply to this Work are set out in our licence referred to above.

Other than as permitted in any relevant BMJ Author's Self Archiving Policies, I confirm this Work has not been accepted for publication elsewhere, is not being considered for publication elsewhere and does not duplicate material already published. I confirm all authors consent to publication of this Work and authorise the granting of this licence.

For peer review only

Title: Implementation of medicines pricing policies in sub-Saharan Africa: protocol for a systematic review

Authors:

- Professor Tolib Mirzoev*, University of Leeds, Nuffield Centre for International Health and Development t.mirzoev@leeds.ac.uk
- Dr Augustina Koduah, University of Ghana, School of Pharmacy akoduah@ug.edu.gh
- Dr Anna Cronin de Chavez, University of Leeds, Nuffield Centre for International Health and Development A.Chavez@leeds.ac.uk
- Dr Leonard Baatiema, University of Ghana, School of Pharmacy baatiemaleonard@gmail.com
- Dr Anthony Danso-Appiah, University of Ghana, School of Public Health adanso-appiah@ug.edu.gh
- Professor Tim Ensor, University of Leeds, Nuffield Centre for International Health and Development T.R.A.Ensor@leeds.ac.uk
- Professor Irene Akua Agyepong, Ghana Health Service, Research and Development Division irene.agyepong@gmail.com
- Mrs Judy Wright, University of Leeds, Leeds Institute of Health Sciences J.M.Wright@leeds.ac.uk
- Dr Irene A Kretchy, University of Ghana, School of Pharmacy ikretchy@ug.edu.gh
- Ms Natalie King, University of Leeds, Leeds Institute of Health Sciences N.V.King@leeds.ac.uk

*Corresponding author: Nuffield Centre for International Health and Development, Level 10 Worsley Building, Clarendon Way, Leeds LS2 9NL. t.mirzoev@leeds.ac.uk +44(0)113-343-4869

Keywords

Systematic review; medicine pricing; policy implementation; sub-Saharan Africa; global health

Word count

2,228 words

For peer review only - http://bmjopen.bmj.com/site/about/guidelines.xhtml

Abstract

Introduction

Ensuring universal availability and accessibility of medicines and supplies is critical for national health systems to equitably address population health needs. In sub-Saharan Africa (SSA), this is a recognised priority with multiple medicines pricing policies enacted. However, medicine prices have remained high, continue to rise and constrain their accessibility. In this systematic review, we aim to identify and analyse experiences of implementation of medicines pricing policies in SSA. Our ambition is for this evidence to contribute to improved implementation of medicines pricing policies in SSA.

Methods and analysis

We will search: Medline, Web of Science, Scopus, Global Health, Embase, Cairn.Info International Edition, Erudit and African Index Medicus, the grey literature and reference from related publications. The searches will be limited to literature published from the year 2000 onwards i.e. since the start of the Millennium Development Goals.

Published peer-reviewed studies of implementation of medicines pricing policies in sub-Saharan Africa will be eligible for inclusion. Broader policy analyses and documented experiences of implementation of other health policies will be excluded. The team will collaboratively screen titles and abstracts, then two reviewers will independently screen full-texts, extract data and assess quality of the included studies. Disagreements will be resolved by discussion or a third reviewer. Data will be extracted on approaches used for policy implementation, actors involved, evidence used in decision-making and key contextual influences on policy implementation. A narrative approach will be used to synthesise the data. Reporting will be informed by the Preferred Reporting Items for Systematic Review and Meta-Analysis Protocols (PRISMA) guideline.

Ethics and dissemination

No ethics approvals are required for systematic reviews.

Results will be disseminated through academic publications, policy briefs and presentations to national policymakers in Ghana and mode widely across countries in SSA.

Registration details

Prospero registration number: CRD42020178166, full record: https://www.crd.york.ac.uk/prospero/display_record.php?ID=CRD42020178166

For peer review only

Article summary

Strengths and limitations of this study

- This systematic review protocol follows the Preferred Reporting Items for Systematic Review and Meta-Analysis Protocols (PRISMA-P) guidelines;
- The review addresses a gap in the current knowledge of the determinants and outcomes of successful implementation of medicines pricing policies in sub-Saharan Africa;
- The focus on sub-Saharan Africa (SSA) will help with transferability of lessons across the different countries within the region, though this may lead to omission of important experiences for example from Asia and Latin America and may limit transferability of lessons outside the SSA;
- The search will be restricted to peer-reviewed published articles and grey literature, thus relevant theses and conference abstracts are likely to be omitted and may affect the depth of evidence on the topic;
- The narrative synthesis approach reflects the nature of published evidence on the topic of policy implementation with no meta-analysis possible, and is a potential limitation of this review.

For peer review only

Introduction

The current agenda of Universal Health Coverage (UHC) highlights the importance of access to safe, quality and affordable medicines as its key driver.[1-3] Increasing access to essential medicines through medicines pricing interventions is an issue of current health policy discourses. [4 5] In response, various policy initiatives have evolved to regulate medicine pricing and improve access.

Globally, different medicine pricing models and strategies exist. These include: generic or biosimilar price linking to originator products, non-proprietary prescribing and generic substitutions, tendering and pooled procurements, internal reference pricing, external price referencing or international price comparisons, and managed-entry agreements. [6-8] Implementation of these medicine pricing policies may be dependent on in-country manufacturing capacity, pricing levels of the medicines, whether medicines are generic or branded, and whether medicines are for out-patient or in-patient services.[9]

Many of these medicine pricing policies are being implemented in high-income countries. However, unlike in high-income countries, low and middle-income countries (LMICs) have less regulated and developed pharmaceutical markets and have different challenges in distribution and production. [1] In light of this, multiple medicine pricing models and strategies are required to achieve equitable access to safe, quality and affordable medicines [10], particularly in sub-Saharan African countries (SSA). [10]

Rationale

Ensuring availability and accessibility of medicines is an important mechanism by which national health systems can equitably address health needs of their populations, including the poorest and the most vulnerable. In SSA, this is a recognised policy priority. For example, in the last two decades different medicines pricing policies were implemented in South Africa [11 12] and between 2012 and 2017, the Government of Ghana introduced four policies to improve access to medicines through medicine price regulation, and ultimately health outcomes and quality of life. These policies are currently at different stages of their implementation and despite these efforts, medicine prices have remained high and continue to rise, making them inaccessible to a large proportion of populations.. This raises questions as to why and how these policies are failing to achieve the desired outcomes.

In this systematic review, we will explore the effectiveness of implementation of medicines pricing policies in the sub-Saharan African context. We want to identify which policies have been implemented and then explore three broad dimensions of their implementation. First, we want to understand *what happened* i.e. identify evidence on effective implementations of medicines pricing policies reflected in a reduction in prices and improvement in access to medicines and subsequently healthcare. Second, we want to understand *how it happened* i.e. we want to identify and unpack the implementation processes and approaches deployed in terms of their timing, participation of actors and role of evidence. Third, we want to understand *why it happened*. We want to identify and synthesise key reported facilitators and barriers to the implementation and understand how they affected the implementation of these policies within their respective contexts.

For peer review only - http://bmjopen.bmj.com/site/about/guidelines.xhtml

Aim and objectives

In this systematic review we will address the following overall question: what are the key determinants of implementation of medicines pricing policies in sub-Saharan African (SSA) countries? More specifically, we will answer four questions:

a. Which medicines pricing policies have been implemented in SSA and what are their key elements?
b. How have these policies been implemented (in relation to implementation approaches, processes, involvement of actors, role of evidence etc)?
c. Which key facilitators and barriers affected the implementation of medicines pricing policies, and how?
d. Which implementation of medicines pricing policies in SSA are effective (in relation to reducing prices of medicines and improving access to services)?

This review is being undertaken during April 2020 – May 2021 as part of the project on 'Improving equitable access to essential medicines in Ghana through bridging the gaps in implementing medicines pricing policy' (AMIPS) – a NIHR-funded award received jointly by the University of Leeds, University of Ghana and the Ghana Health Service. The results of this review will be combined with results of policy analyses in Ghana and will inform engagements with key stakeholders on improving the implementation of the current policies and identification of future research and development priorities. Our ambition is for evidence from this review to contribute to improved implementation of medicines pricing policies across countries of SSA.

This protocol follows the Preferred reporting items for systematic review and meta-analysis protocols (PRISMA-P) guidelines[13] and a PRISMA-P checklist is available as a supplementary file.

Methods and analysis

Eligibility criteria

Studies

We will include empirical studies including Randomised Controlled Trials (RCTs), quasi-experimental studies and cohort and cross-sectional studies. Reviews (scoping reviews, meta-syntheses, realist syntheses) will also be included and individual primary studies from the systematic reviews will be manually included as empirical literature. We will exclude opinion pieces and conceptual/theoretical publications which do not report documented empirical data from either primary studies or reviews.

Specific inclusion criteria will be: (a) focus on the medicines pricing policies i.e. policies, strategies, interventions or plans which aim to improve affordability of medicines in the country. The link to improvements in access to healthcare may be implicit and is not a requirement; (b) focus on policy implementation i.e. either as part of the whole policy process (agenda-setting, development, implementation) or as an exclusive focus; (c) sub-Saharan African country contexts i.e. either as part of the comparative studies or as a sole focus; (d) studies which were published since the agenda of Millennium Development Goals was initiated shortly before 2000; and (e) papers with relevant information available for analysis.

Specific exclusion criteria are: (a) policy analyses which focus solely on policy agenda-setting and development stages of the policy process; (b) studies from high-income country contexts and outside sub-Saharan Africa; (c) studies conducted two years or more prior to 2000 but published after 2000, will be excluded in consideration of MDG and SDG agenda which started in 2000; (d)

For peer review only - http://bmjopen.bmj.com/site/about/guidelines.xhtml

papers in languages where we are unable to have the resources for translation (the team has access to French, Spanish and Russian-speaking researchers) and (e) papers with no full text available for analysis.

Participants

The participants to be covered in this review will be: policymakers, implementers, service providers, patients and beneficiaries of successful implementation of medicines pricing policies (of any gender, age, ethnicity, socio-economic group, health status or urban-rural residence).

Interventions

Implementation of medicines pricing policies i.e. policies, strategies, interventions or plans which aim to improve affordability of medicines in the country.

Comparison

No comparison or control is applicable to this study

Outcomes

Successful implementation will be measured as reduction in medicines prices, and improved access to medicines along the supply chain. Any studies describing unsuccessful implementation will also be used to inform the lessons learned.

Study records

Searches

We will search the following databases: Medline (1946 – present), Web of Science (1990 – present), Scopus (1823 – present), Global Health (1973 – present), Embase (1947 – present), Cairn.Info International Edition (all available years), Erudit (all available years) and African Index Medicus (all available years). The Medline search strategy is available as a supplementary file. The search strategies will incorporate index terms (MeSH) and text words for the search concepts:

1. Sub-Saharan African Countries. This will include terms/synonyms for sub-Saharan Africa AND list of individual countries in the region
2. Drug/Medicines pricing. This will include terms/synonyms for: medicines / pharmaceuticals / drugs / prescriptions AND pricing / cost / affordability / fees / purchase / rebate(s) / tariffs / incentives / benchmarking / reference pricing / payment / spend* / expenditure / subsid* / procurement
3. Policy. This will include terms/synonyms for: policy / strategy / plan / framework / regulations / guidelines / rules / intervention / tax / exemption
4. Implementation. This will include terms / synonyms for: Implementing / Implement(s) / Implementation approach(es) / Process(es) / Facilitator(s) / Barrier(s) / Factors / Determinants / context

The searches will be limited to the literature published from the year 2000 and onwards. This is in consideration of the Millennium Development Goals agenda which started in 2000 with a clear focus on improving access to medicines and services. We will follow-up on the references to the individual studies as required. We will manually search for the included references in relevant retrieved reviews (systematic reviews, scoping reviews, meta-syntheses, realist syntheses) for additional

For peer review only - http://bmjopen.bmj.com/site/about/guidelines.xhtml

relevant studies for inclusion. In addition, we will search grey literature including global development websites: WHO IRIS, World Bank, DFID/K4D repository, Gates Foundation and contacts with experts in the field.

Data management

We will upload all references identified through searches (electronic database and additional searches) into Endnote version X9. Once duplicates are removed, the remaining references will be exported into Rayyan (https://rayyan.qcri.org/welcome), an online free systematic review tool for screening.

Screening

Titles and abstracts will be divided up across the review team and screened individually for eligibility using pre-specified eligibility criteria flowchart, which is available in a supplementary file. At least 20% of individually reviewed titles and abstracts will then be cross-checked by at least two members of the team. Full texts will be obtained for all the potentially relevant studies and screened by two members of the team independently, and disagreements will be resolved through discussion. Where necessary, a third member of the team will engage to help resolve disagreements.

Data extraction

The following data will be extracted by two members of the review team into an appropriate data extraction form:

- article information (full citation, year study was conducted, study type, setting / country);
- medicine pricing policies studied (including which key elements the policies included);
- documented effects on prices of medicines (including how identified and reported);
- effects on access to medicines (including how identified and reported);
- effects on access to healthcare (including how identified and reported);
- implementation approach (including processes, actors involved and their roles and evidence used to inform implementation);
- key influences on policy implementation (including facilitators and constraints and how they affected implementation).

Quality assessment and risk of bias

Quality of each included study will be appraised. We will utilise validated quality assessment tools and the critical appraisal tools for relevant studies (qualitative and quantitative research) from the Joanna Briggs Institute https://joannabriggs.org/ebp/critical_appraisal_tools. While at this point, we do not intend to change the actual criteria, the interpretation and application of the tools will be within the context of our study which focuses on key determinants of effective implementation of medicines pricing policies in SSA context. For example, clarity of focus will be assessed in relation to how the different aspects of policy implementation (processes, use of evidence, involvement of actors) are identified and consistently used in the reviewed papers.

A careful assessment of risk of bias in the included studies will be performed by two reviewers, who will first independently assess the quality of each study against each criterion. Results will be shared and agreed, and any disagreements will be addressed through engaging a third reviewer.

For peer review only - http://bmjopen.bmj.com/site/about/guidelines.xhtml

Data synthesis and interpretation

Strategy for data synthesis.

The main outcome in our study is the medicine pricing policy implementation. Policy implementation is typically done within a single country, but where the same policy is implemented in different countries, the analysis will take the specific context of the country into consideration.

In exploring the policy implementation, we will employ established policy theories and frameworks such as Walt and Gilson's policy triangle [14], Baumgartner's punctuated equilibrium [15] and Lipsky's street-level bureaucracy [16], and will also draw upon further theories and frameworks developed or adapted within the reviewed papers.

Where possible, we will compare the effects of the policies in a quantitative synthesis. We anticipate, however, that the heterogeneity of reporting of outcomes and of context may make it impossible to conduct a meta-analysis. In such a situation we will focus on narrative synthesis.

Whilst using qualitative or narrative synthesis approach [17], data related to the medicines pricing policies will be extracted from the Introduction, Methods and possibly Results sections. Data on the effects of policy implementation and the policy implementation approaches, and key influences will be extracted from the Results and Discussion sections. Extracted data will be analysed thematically and will be structured around the specific questions of the review.

The interpretation of the results will follow the identified themes for each review question. For example, in answering the third review question we will divide the factors into facilitators and constraints and potentially will further sub-divide them by their nature (e.g. community issues, health systems issues, wider socio-economic influences).

At the moment, we are not planning analysis of sub-groups or sub-sets. However, depending on the breadth of extracted data we may consider sub-groups such as geographical region (West Africa, East Africa, Southern Africa), setting (urban, rural) or categories of implementers (health facilities, pharmacies).

Patient and public involvement

No patient involved.

Ethics and dissemination

Ethics approvals are not required for systematic reviews. However, ethics approvals for the wider AMIPS study within which this review is being undertaken have been granted by the ethics committees from the Ghana Health Service (ref GHS-ERC006/02/20) and the University of Leeds School of Medicine (ref MREC 19-060).

We will disseminate results through academic papers and stakeholder workshops in Ghana and other SSA countries where possible. In Ghana, the review results will be complemented by reviews of policy documents. The findings of this review will also be presented at scientific conferences such as the biannual Global Symposia on Health Systems Research and Thematic Working Groups of the Health Systems Global.

The results of this review will inform empirical investigations of implementation of medicines pricing policies in Ghana through the in-depth interviews and focus groups, and engagements and

For peer review only - http://bmjopen.bmj.com/site/about/guidelines.xhtml

consultations with policymakers on seeking ways of further improving the implementation of medicines pricing policies in Ghana.

Authors' contributions

AK and TM jointly conceived the study; TM, AK, AC, LB, ADA, TE, IA, JW, IK, NK contributed to the review design and jointly wrote the protocol; TM, AK, AC, LB, ADA, TE, IA, JW, IK, NK read and approved the final version of the manuscript. AK and TM will be the guarantors of the review.

Funding

This research was commissioned by the National Institute for Health Research (NIHR) NIHR Global Health Policy and Systems Research Development Award using UK aid from the UK Government (grant number 130219). The views expressed in this publication are those of the author(s) and not necessarily those of the NIHR or the Department of Health and Social Care

Competing interests

None declared

References

1. Bigdeli M, Laing R, Tomson G. Medicines and universal health coverage: challenges and opportunities: BioMed Central, 2015. 1-3
2. Ghebreyesus TA. All roads lead to universal health coverage. *The Lancet Global Health* 2017;5(9):e839-e40.
3. Wirtz VJ, Hogerzeil HV, Gray AL, et al. Essential medicines for universal health coverage. *The Lancet* 2017;389(10067):403-76.
4. World Health O, Regional Office for E. Access to new medicines in Europe : technical review of policy initiatives and opportunities for collaboration and research. 2015
5. World Health Organization. Assessment of medicine pricing and reimbursement systems in health insurance schemes. 2016
6. Bashaar M, Hassali F, ALrasheedy A, et al. Efficacy of international approaches to medicine price regulation and control: A scoping review. *J Appl Pharm Sci* 2017;7(04):227-41.
7. Leopold C, Vogler S, Mantel-Teeuwisse AK, et al. Differences in external price referencing in Europe—a descriptive overview. *Health Policy* 2012;104(1):50-60.
8. Vogler S, Paris V, Ferrario A, et al. How can pricing and reimbursement policies improve affordable access to medicines? Lessons learned from European countries. *Applied health economics and health policy* 2017;15(3):307-21.
9. Vogler S, Zimmermann N, de Joncheere K. Policy interventions related to medicines: Survey of measures taken in European countries during 2010–2015. *Health Policy* 2016;120(12):1363-77.

For peer review only - http://bmjopen.bmj.com/site/about/guidelines.xhtml

10. Nguyen TA, Knight R, Roughead EE, et al. Policy options for pharmaceutical pricing and purchasing: issues for low-and middle-income countries. *Health policy and planning* 2015;30(2):267-80.
11. Moodley R, Suleman F. Evaluating the impact of the single exit price policy on a basket of originator medicines in South Africa from 1999 to 2014 using a time series analysis. *BMC Health Services Research* 2019;19(1):576. doi: 10.1186/s12913-019-4403-8
12. Steyn R, Burger JR, Serfontein JHP, et al. Influence of a new reference-based pricing system in South Africa on the prevalence and cost of antidiabetic medicine: a pilot study. *International Journal of Pharmacy Practice* 2007;15(4):307-11. doi: 10.1211/ijpp.15.4.0009
13. Shamseer L, Moher D, Clarke M, et al. Preferred reporting items for systematic review and meta-analysis protocols (PRISMA-P) 2015: elaboration and explanation. *BMJ : British Medical Journal* 2015;349:g7647. doi: 10.1136/bmj.g7647
14. Walt G, Gilson L. Reforming the health sector in developing countries: the central role of policy analysis. *Health Policy Plan* 1994;9(4):353-70.
15. Baumgartner F, Jones B. Agendas and Instability in American Politics. Chicago, London: University of Chicago Press 1993.
16. Lipsky M. Street-level bureaucracy: dilemmas of the individual in public services New York: Russel Sage Foundation 1980.
17. Snilstveit B, Oliver S, Vojtkova M. Narrative approaches to systematic review and synthesis of evidence for international development policy and practice. *Journal of Development Effectiveness* 2012;4(3):409-29. doi: 10.1080/19439342.2012.710641

For peer review only

For peer review only - http://bmjopen.bmj.com/site/about/guidelines.xhtml

Title: Implementation of medicines pricing policies in sub-Saharan Africa: protocol for a systematic review

PRISMA-P (preferred reporting items for systematic review and meta-analysis protocols) 2015 checklist: recommended items to address in a systematic review protocol

Section and topic	*Item No*	*Checklist item*	*Y/N*
**Administrative information**			
Title:			
Identification	1a	Identify the report as a protocol of a systematic review	Y
Update	1b	If the protocol is for an update of a previous systematic review, identify as such	Y
Registration	2	If registered, provide the name of the registry (such as PROSPERO) and registration number	Y
Authors:			
Contact	3a	Provide name, institutional affiliation, e-mail address of all protocol authors; provide physical mailing address of corresponding author	Y
Contributions	3b	Describe contributions of protocol authors and identify the guarantor of the review	Y
Amendments	4	If the protocol represents an amendment of a previously completed or published protocol, identify as such and list changes; otherwise, state plan for documenting important protocol amendments	n/a
Support:			
Sources	5a	Indicate sources of financial or other support for the review	Y
Sponsor	5b	Provide name for the review funder and/or sponsor	Y
Role of sponsor or funder	5c	Describe roles of funder(s), sponsor(s), and/or institution(s), if any, in developing the protocol	Y
**Introduction**			
Rationale	6	Describe the rationale for the review in the context of what is already known	Y
Objectives	7	Provide an explicit statement of the question(s) the review will address with reference to participants, interventions, comparators, and outcomes (PICO)	Y
**Methods**			
Eligibility criteria	8	Specify the study characteristics (such as PICO, study design, setting, time frame) and report characteristics (such as years considered, language, publication status) to be used as criteria for eligibility for the review	Y
Information sources	9	Describe all intended information sources (such as electronic databases, contact with study authors, trial registers or other grey literature sources) with planned dates of coverage	Y
Search strategy	10	Present draft of search strategy to be used for at least one electronic database, including planned limits, such that it could be repeated	Y
Study records:			

For peer review only

Section and topic	*Item No*	*Checklist item*	*Y/N*
Data management	11a	Describe the mechanism(s) that will be used to manage records and data throughout the review	Y
Selection process	11b	State the process that will be used for selecting studies (such as two independent reviewers) through each phase of the review (that is, screening, eligibility and inclusion in meta-analysis)	Y
Data collection process	11c	Describe planned method of extracting data from reports (such as piloting forms, done independently, in duplicate), any processes for obtaining and confirming data from investigators	Y
Data items	12	List and define all variables for which data will be sought (such as PICO items, funding sources), any pre-planned data assumptions and simplifications	Y
Outcomes and prioritization	13	List and define all outcomes for which data will be sought, including prioritization of main and additional outcomes, with rationale	Y
Risk of bias in individual studies	14	Describe anticipated methods for assessing risk of bias of individual studies, including whether this will be done at the outcome or study level, or both; state how this information will be used in data synthesis	Y
Data synthesis	15a	Describe criteria under which study data will be quantitatively synthesised	Y
	15b	If data are appropriate for quantitative synthesis, describe planned summary measures, methods of handling data and methods of combining data from studies, including any planned exploration of consistency (such as I2, Kendall's τ)	n/a
	15c	Describe any proposed additional analyses (such as sensitivity or subgroup analyses, meta-regression)	n/a
	15d	If quantitative synthesis is not appropriate, describe the type of summary planned	Y
Meta-bias(es)	16	Specify any planned assessment of meta-bias(es) (such as publication bias across studies, selective reporting within studies)	Y
Confidence in cumulative evidence	17	Describe how the strength of the body of evidence will be assessed (such as GRADE)	Y

For peer review only

Along with Medline we intend to search the following databases:

African Index Medicus (via WHO Global Health Index Medicus) all available years; Embase (Ovid) 1996 present; Global Health (Ovid) 1973 to present; Scopus (Elsevier B.V.) 1823 – Present; Web of Science Core Collection: Citation Indexes (Clarivate Analytics) 1900-present;. We will also search for grey literature and French articles in the following; Cairn International (Cairn Info) all available years; Erudit (University of Montreal ???) all available years; IRIS Institutional Repository for Information Sharing (WHO) all available years and World Bank Group Research and Publications

Sample Medline strategy

Ovid MEDLINE(R) and Epub Ahead of Print, In-Process & Other Non-Indexed Citations and Daily <1946 to April 23, 2020>

Search Strategy:

----

1 exp "Africa South of the Sahara"/ (205870)

2 (angola* or benin* or botswana* or "burkina faso" or burundi*).ti,ab,in,kf. (17906)

3 ("cabo verde*" or "cape verde*" or cameroon* or "central africa*" or chad or cormoros or congo* or "ivory coast" or "cote d'ivoire" or djibouti).ti,ab,in,kf. (45277)

4 (guinea* or eritrea* or eswatini* or swaziland* or ethiopia* or gabon* or gambia* or ghana* or guinea*).ti,ab,in,kf. (158364)

5 (kenya* or lesotho* or liberia* or madagasca* or malawi* or mali or mauritania* or mauritius or mozambique*).ti,ab,in,kf. (55553)

6 (namibia* or niger or nigeria* or rwanda*).ti,ab,in,kf. (67659)

7 ("sao tome" or principe* or senegal* or seychelles or "sierra leone*" or somali* or "south africa*" or sudan*).ti,ab,in,kf. (132358)

8 (tanzania* or togo* or uganda* or zambia* or zaire* or zimbabw*).ti,ab,in,kf. (50798)

9 (africa* adj2 ("sub sahara*" or "south* sahara*")).ti,ab,in,kf. (23957)

10 or/1-9 [sub-saharan africa] (519682)

11 Drug Costs/ (15924)

12 ((price? or pricing) adj5 (medicine? or drug? or prescription? or pharmaceutical*)).tw,kw. (4558)

13 ((cost or costs) adj5 (medicine? or drug? or prescription? or pharmaceutical*)).tw,kw. (18930)

14 (afford* adj5 (medicine? or drug? or prescription? or pharmaceutical*)).tw,kw. (1929)

15 (reimburs* adj5 (medicine? or drug? or prescription? or pharmaceutical*)).tw,kw. (2099)

16 (generic* adj5 (medicine? or drug? or prescription? or pharmaceutical*)).tw,kw. (4521)

17 exp fees, pharmaceutical/ (2413)

For peer review only

18 ((purchas* or procur* or expenditure*) adj5 (medicine? or drug? or prescription? or pharmaceutical*)).tw,kw. (4913)

19 ((subsid* or tariff* or incentive* or containment or transparency) adj5 (medicine? or drug? or prescription? or pharmaceutical*)).tw,kw. (2141)

20 ((fee or fees or rebate* or payment* or spend* or saving*) adj5 (medicine? or drug? or prescription? or pharmaceutical*)).tw,kw. (4006)

21 ((benchmark* or cost-plus) adj12 (medicine? or drug? or prescription? or pharmaceutical*)).tw,kw. (641)

22 "low* price* generic*".tw,kw. (76)

23 (essential adj2 (drug* or medicine*)).tw,kw. (3434)

24 Drugs, Essential/ (835)

25 (access* adj3 (medicine? or drug? or prescription? or pharmaceutical*)).tw,kw. (5991)

26 Economics, Pharmaceutical/ (2927)

27 (pharma* adj2 economic*).tw,kw. (833)

28 pharmacoeconomic*.tw,kw. (3898)

29 or/11-28 [drug pricing] (58841)

30 exp policy/ (155119)

31 Government Regulation/ (21073)

32 exp Legislation, Drug/ (32091)

33 ((drug* or medicine* or pharmaceutical* or prescription* or health*) adj7 (guideline* or guidance or policy or policies or law or regulat* or rule* or legislat* or control* or strateg* or framework*)).tw,kw. (538082)

34 (tax or taxes or exemption*).tw,kw. (17307)

35 ((drug* or medicine* or pharmaceutical* or prescription* or health*) adj7 (intervention* or plan* or program*)).tw,kw. (256052)

36 or/30-35 [policy concept -all] (917754)

37 10 and 29 and 36 [SSA and policy] (1190)

38 Health Plan Implementation/ (5829)

39 Program Evaluation/ (62240)

40 (barrier* or facilitator* or challenge* or motivator*).tw,kw. (923252)

41 (implement* or approach* or process*).tw,kw. (3962356)

42 (factor* or determinant* or context*).tw,kw. (3829825)

43 "scal* up".tw,kw. (19228)

For peer review only

44 adopt*.tw,kw. (243593)

45 or/38-44 [implementation] (7698904)

46 10 and 29 and 36 and 45 (763)

47 limit 46 to yr="2000 -Current" (652)

For peer review only

AMIPS project: Flowchart for screening of search results

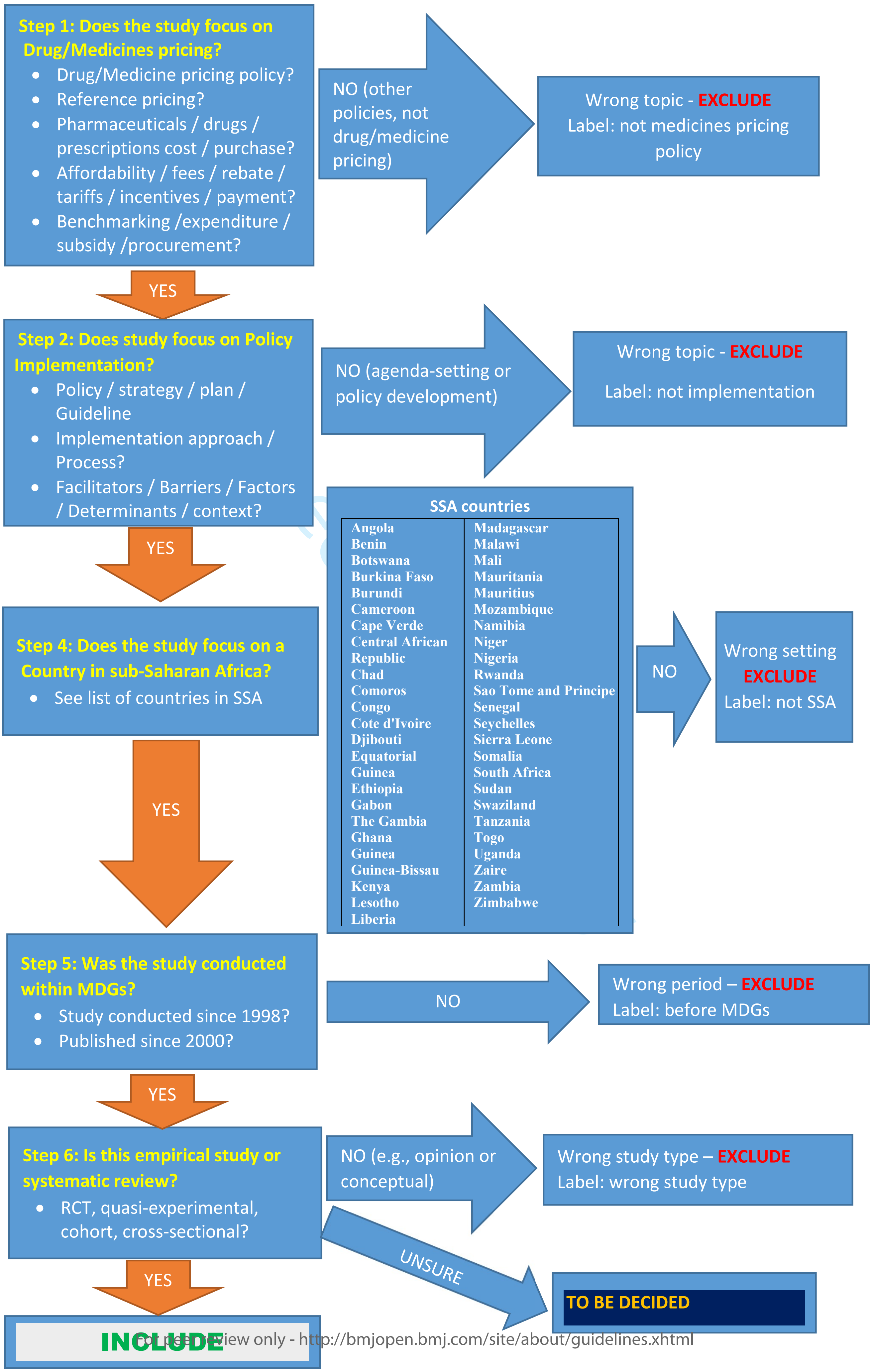

For peer review only - http://bmjopen.bmj.com/site/about/guidelines.xhtml

BMJ Open

Implementation of medicines pricing policies in sub-Saharan Africa: protocol for a systematic review

Journal:	*BMJ Open*
Manuscript ID	bmjopen-2020-044293.R2
Article Type:	Protocol
Date Submitted by the Author:	16-Jan-2021
Complete List of Authors:	Mirzoev, Tolib; University of Leeds, Nuffield Centre for International Health and Development<br>Koduah, Augustina ; University of Ghana, School of Pharmacy<br>Cronin de Chavez, Anna; University of Leeds, Nuffield Centre for International Health and Development<br>Baatiema, Leonard; University of Ghana, School of Pharmacy<br>Danso-Appiah, Anthony; University of Ghana, School of Public Health<br>Ensor, Tim; University of Leeds, Nuffield Centre for International Health and Development<br>Agyepong, Irene; Ghana Health Service, Research and Development Division<br>Wright, Judy; University of Leeds, Leeds Institute of Health Sciences<br>Kretchy, Irene ; University of Ghana, School of Pharmacy<br>King, Natalie; University of Leeds, Leeds Institute of Health Sciences
**Primary Subject Heading**:	Health policy
Secondary Subject Heading:	Global health
Keywords:	Health policy < HEALTH SERVICES ADMINISTRATION & MANAGEMENT, International health services < HEALTH SERVICES ADMINISTRATION & MANAGEMENT, PUBLIC HEALTH

SCHOLARONE™
Manuscripts

I, the Submitting Author has the right to grant and does grant on behalf of all authors of the Work (as defined in the below author licence), an exclusive licence and/or a non-exclusive licence for contributions from authors who are: i) UK Crown employees; ii) where BMJ has agreed a CC-BY licence shall apply, and/or iii) in accordance with the terms applicable for US Federal Government officers or employees acting as part of their official duties; on a worldwide, perpetual, irrevocable, royalty-free basis to BMJ Publishing Group Ltd ("BMJ") its licensees and where the relevant Journal is co-owned by BMJ to the co-owners of the Journal, to publish the Work in this journal and any other BMJ products and to exploit all rights, as set out in our licence.

The Submitting Author accepts and understands that any supply made under these terms is made by BMJ to the Submitting Author unless you are acting as an employee on behalf of your employer or a postgraduate student of an affiliated institution which is paying any applicable article publishing charge ("APC") for Open Access articles. Where the Submitting Author wishes to make the Work available on an Open Access basis (and intends to pay the relevant APC), the terms of reuse of such Open Access shall be governed by a Creative Commons licence – details of these licences and which Creative Commons licence will apply to this Work are set out in our licence referred to above.

Other than as permitted in any relevant BMJ Author's Self Archiving Policies, I confirm this Work has not been accepted for publication elsewhere, is not being considered for publication elsewhere and does not duplicate material already published. I confirm all authors consent to publication of this Work and authorise the granting of this licence.

For peer review only

Title: Implementation of medicines pricing policies in sub-Saharan Africa: protocol for a systematic review

Authors:

- Professor Tolib Mirzoev*, University of Leeds, Nuffield Centre for International Health and Development t.mirzoev@leeds.ac.uk
- Dr Augustina Koduah, University of Ghana, School of Pharmacy akoduah@ug.edu.gh
- Dr Anna Cronin de Chavez, University of Leeds, Nuffield Centre for International Health and Development A.Chavez@leeds.ac.uk
- Dr Leonard Baatiema, University of Ghana, School of Pharmacy baatiemaleonard@gmail.com
- Dr Anthony Danso-Appiah, University of Ghana, School of Public Health adanso-appiah@ug.edu.gh
- Professor Tim Ensor, University of Leeds, Nuffield Centre for International Health and Development T.R.A.Ensor@leeds.ac.uk
- Professor Irene Akua Agyepong, Ghana Health Service, Research and Development Division irene.agyepong@gmail.com
- Mrs Judy Wright, University of Leeds, Leeds Institute of Health Sciences J.M.Wright@leeds.ac.uk
- Dr Irene A Kretchy, University of Ghana, School of Pharmacy ikretchy@ug.edu.gh
- Ms Natalie King, University of Leeds, Leeds Institute of Health Sciences N.V.King@leeds.ac.uk

*Corresponding author: Nuffield Centre for International Health and Development, Level 10 Worsley Building, Clarendon Way, Leeds LS2 9NL. t.mirzoev@leeds.ac.uk +44(0)113-343-4869

Keywords

Systematic review; medicine pricing; policy implementation; sub-Saharan Africa; global health

Word count

2,228 words

For peer review only - http://bmjopen.bmj.com/site/about/guidelines.xhtml

Abstract

Introduction

Ensuring universal availability and accessibility of medicines and supplies is critical for national health systems to equitably address population health needs. In sub-Saharan Africa (SSA), this is a recognised priority with multiple medicines pricing policies enacted. However, medicine prices have remained high, continue to rise and constrain their accessibility. In this systematic review, we aim to identify and analyse experiences of implementation of medicines pricing policies in SSA. Our ambition is for this evidence to contribute to improved implementation of medicines pricing policies in SSA.

Methods and analysis

We will search: Medline, Web of Science, Scopus, Global Health, Embase, Cairn.Info International Edition, Erudit and African Index Medicus, the grey literature and reference from related publications. The searches will be limited to literature published from the year 2000 onwards i.e. since the start of the Millennium Development Goals.

Published peer-reviewed studies of implementation of medicines pricing policies in sub-Saharan Africa will be eligible for inclusion. Broader policy analyses and documented experiences of implementation of other health policies will be excluded. The team will collaboratively screen titles and abstracts, then two reviewers will independently screen full-texts, extract data and assess quality of the included studies. Disagreements will be resolved by discussion or a third reviewer. Data will be extracted on approaches used for policy implementation, actors involved, evidence used in decision-making and key contextual influences on policy implementation. A narrative approach will be used to synthesise the data. Reporting will be informed by the Preferred Reporting Items for Systematic Review and Meta-Analysis Protocols (PRISMA) guideline.

Ethics and dissemination

No ethics approvals are required for systematic reviews.

Results will be disseminated through academic publications, policy briefs and presentations to national policymakers in Ghana and mode widely across countries in SSA.

Registration details

Prospero registration number: CRD42020178166, full record: https://www.crd.york.ac.uk/prospero/display_record.php?ID=CRD42020178166

For peer review only - http://bmjopen.bmj.com/site/about/guidelines.xhtml

Article summary

Strengths and limitations of this study

- This systematic review protocol follows the Preferred Reporting Items for Systematic Review and Meta-Analysis Protocols (PRISMA-P) guidelines;
- The review addresses a gap in the current knowledge of the determinants and outcomes of successful implementation of medicines pricing policies in sub-Saharan Africa;
- The focus on sub-Saharan Africa (SSA) will help with transferability of lessons across the different countries within the region, though this may lead to omission of important experiences for example from Asia and Latin America and may limit transferability of lessons outside the SSA;
- The search will be restricted to peer-reviewed published articles and grey literature, thus relevant theses and conference abstracts are likely to be omitted and may affect the depth of evidence on the topic;
- The narrative synthesis approach reflects the nature of published evidence on the topic of policy implementation with no meta-analysis possible, and is a potential limitation of this review.

For peer review only

For peer review only - http://bmjopen.bmj.com/site/about/guidelines.xhtml

Introduction

The current agenda of Universal Health Coverage (UHC) highlights the importance of access to safe, quality and affordable medicines as its key driver.[1-3] Increasing access to essential medicines through medicines pricing interventions is an issue of current health policy discourses. [4 5] In response, various policy initiatives have evolved to regulate medicine pricing and improve access.

Globally, different medicine pricing models and strategies exist. These include: generic or biosimilar price linking to originator products, non-proprietary prescribing and generic substitutions, tendering and pooled procurements, internal reference pricing, external price referencing or international price comparisons, and managed-entry agreements. [6-8] Implementation of these medicine pricing policies may be dependent on in-country manufacturing capacity, pricing levels of the medicines, whether medicines are generic or branded, and whether medicines are for out-patient or in-patient services.[9]

Many of these medicine pricing policies are being implemented in high-income countries. However, unlike in high-income countries, low and middle-income countries (LMICs) have less regulated and developed pharmaceutical markets and have different challenges in distribution and production. [1] In light of this, multiple medicine pricing models and strategies are required to achieve equitable access to safe, quality and affordable medicines [10], particularly in sub-Saharan African countries (SSA). [10]

Rationale

Ensuring availability and accessibility of medicines is an important mechanism by which national health systems can equitably address health needs of their populations, including the poorest and the most vulnerable. In SSA, this is a recognised policy priority. For example, in the last two decades different medicines pricing policies were implemented in South Africa [11 12] and between 2012 and 2017, the Government of Ghana introduced four policies to improve access to medicines through medicine price regulation, and ultimately health outcomes and quality of life. These policies are currently at different stages of their implementation and despite these efforts, medicine prices have remained high and continue to rise, making them inaccessible to a large proportion of populations.. This raises questions as to why and how these policies are failing to achieve the desired outcomes.

In this systematic review, we will explore the effectiveness of implementation of medicines pricing policies in the sub-Saharan African context. We want to identify which policies have been implemented and then explore three broad dimensions of their implementation. First, we want to understand *what happened* i.e. identify evidence on effective implementations of medicines pricing policies reflected in a reduction in prices and improvement in access to medicines and subsequently healthcare. Second, we want to understand *how it happened* i.e. we want to identify and unpack the implementation processes and approaches deployed in terms of their timing, participation of actors and role of evidence. Third, we want to understand *why it happened*. We want to identify and synthesise key reported facilitators and barriers to the implementation and understand how they affected the implementation of these policies within their respective contexts.

For peer review only - http://bmjopen.bmj.com/site/about/guidelines.xhtml

Aim and objectives

In this systematic review we will address the following overall question: what are the key determinants of implementation of medicines pricing policies in sub-Saharan African (SSA) countries? More specifically, we will answer four questions:

a. Which medicines pricing policies have been implemented in SSA and what are their key elements?
b. How have these policies been implemented (in relation to implementation approaches, processes, involvement of actors, role of evidence etc)?
c. Which key facilitators and barriers affected the implementation of medicines pricing policies, and how?
d. Which implementation of medicines pricing policies in SSA are effective (in relation to reducing prices of medicines and improving access to services)?

This review is being undertaken during April 2020 – May 2021 as part of the project on 'Improving equitable access to essential medicines in Ghana through bridging the gaps in implementing medicines pricing policy' (AMIPS) – a NIHR-funded award received jointly by the University of Leeds, University of Ghana and the Ghana Health Service. The results of this review will be combined with results of policy analyses in Ghana and will inform engagements with key stakeholders on improving the implementation of the current policies and identification of future research and development priorities. Our ambition is for evidence from this review to contribute to improved implementation of medicines pricing policies across countries of SSA.

This protocol follows the Preferred reporting items for systematic review and meta-analysis protocols (PRISMA-P) guidelines[13] and a PRISMA-P checklist is available as a supplementary file.

Methods and analysis

Eligibility criteria

Studies

We will include empirical studies including Randomised Controlled Trials (RCTs), quasi-experimental studies and cohort and cross-sectional studies. Reviews (scoping reviews, meta-syntheses, realist syntheses) will also be included and individual primary studies from the systematic reviews will be manually included as empirical literature. We will exclude opinion pieces and conceptual/theoretical publications which do not report documented empirical data from either primary studies or reviews.

Specific inclusion criteria will be: (a) focus on the medicines pricing policies i.e. policies, strategies, interventions or plans which aim to improve affordability of medicines in the country. The link to improvements in access to healthcare may be implicit and is not a requirement; (b) focus on policy implementation i.e. either as part of the whole policy process (agenda-setting, development, implementation) or as an exclusive focus; (c) sub-Saharan African country contexts i.e. either as part of the comparative studies or as a sole focus; (d) studies which were published since the agenda of Millennium Development Goals was initiated shortly before 2000; and (e) papers with relevant information available for analysis.

Specific exclusion criteria are: (a) policy analyses which focus solely on policy agenda-setting and development stages of the policy process; (b) studies from high-income country contexts and outside sub-Saharan Africa; (c) studies conducted two years or more prior to 2000 but published after 2000, will be excluded in consideration of MDG and SDG agenda which started in 2000; (d)

For peer review only - http://bmjopen.bmj.com/site/about/guidelines.xhtml

papers in languages where we are unable to have the resources for translation (the team has access to French, Spanish and Russian-speaking researchers) and (e) papers with no full text available for analysis.

Participants

The participants to be covered in this review will be: policymakers, implementers, service providers, patients and beneficiaries of successful implementation of medicines pricing policies (of any gender, age, ethnicity, socio-economic group, health status or urban-rural residence).

Interventions

Implementation of medicines pricing policies i.e. policies, strategies, interventions or plans which aim to improve affordability of medicines in the country.

Comparison

No comparison or control is applicable to this study

Outcomes

Successful implementation will be measured as reduction in medicines prices, and improved access to medicines along the supply chain. Any studies describing unsuccessful implementation will also be used to inform the lessons learned.

Study records

Searches

We will search the following databases: Medline (1946 – present), Web of Science (1990 – present), Scopus (1823 – present), Global Health (1973 – present), Embase (1947 – present), Cairn.Info International Edition (all available years), Erudit (all available years) and African Index Medicus (all available years). The Medline search strategy is available as a supplementary file. The search strategies will incorporate index terms (MeSH) and text words for the search concepts:

1. Sub-Saharan African Countries. This will include terms/synonyms for sub-Saharan Africa AND list of individual countries in the region
2. Drug/Medicines pricing. This will include terms/synonyms for: medicines / pharmaceuticals / drugs / prescriptions AND pricing / cost / affordability / fees / purchase / rebate(s) / tariffs / incentives / benchmarking / reference pricing / payment / spend* / expenditure / subsid* / procurement
3. Policy. This will include terms/synonyms for: policy / strategy / plan / framework / regulations / guidelines / rules / intervention / tax / exemption
4. Implementation. This will include terms / synonyms for: Implementing / Implement(s) / Implementation approach(es) / Process(es) / Facilitator(s) / Barrier(s) / Factors / Determinants / context

The searches will be limited to the literature published from the year 2000 and onwards. This is in consideration of the Millennium Development Goals agenda which started in 2000 with a clear focus on improving access to medicines and services. We will follow-up on the references to the individual studies as required. We will manually search for the included references in relevant retrieved reviews (systematic reviews, scoping reviews, meta-syntheses, realist syntheses) for additional

For peer review only - http://bmjopen.bmj.com/site/about/guidelines.xhtml

relevant studies for inclusion. In addition, we will search grey literature including global development websites: WHO IRIS, World Bank, DFID/K4D repository, Gates Foundation and contacts with experts in the field.

Data management

We will upload all references identified through searches (electronic database and additional searches) into Endnote version X9. Once duplicates are removed, the remaining references will be exported into Rayyan (https://rayyan.qcri.org/welcome), an online free systematic review tool for screening.

Screening

Titles and abstracts will be divided up across the review team and screened individually for eligibility using pre-specified eligibility criteria flowchart, which is available in a supplementary file. At least 20% of individually reviewed titles and abstracts will then be cross-checked by at least two members of the team. Full texts will be obtained for all the potentially relevant studies and screened by two members of the team independently, and disagreements will be resolved through discussion. Where necessary, a third member of the team will engage to help resolve disagreements.

Data extraction

The following data will be extracted by two members of the review team into an appropriate data extraction form:

- article information (full citation, year study was conducted, study type, setting / country);
- medicine pricing policies studied (including which key elements the policies included);
- documented effects on prices of medicines (including how identified and reported);
- effects on access to medicines (including how identified and reported);
- effects on access to healthcare (including how identified and reported);
- implementation approach (including processes, actors involved and their roles and evidence used to inform implementation);
- key influences on policy implementation (including facilitators and constraints and how they affected implementation).

Quality assessment and risk of bias

Quality of each included study will be appraised. We will utilise validated quality assessment tools and the critical appraisal tools for relevant studies (qualitative and quantitative research) from the Joanna Briggs Institute https://joannabriggs.org/ebp/critical_appraisal_tools. While at this point, we do not intend to change the actual criteria, the interpretation and application of the tools will be within the context of our study which focuses on key determinants of effective implementation of medicines pricing policies in SSA context. For example, clarity of focus will be assessed in relation to how the different aspects of policy implementation (processes, use of evidence, involvement of actors) are identified and consistently used in the reviewed papers.

A careful assessment of risk of bias in the included studies will be performed by two reviewers, who will first independently assess the quality of each study against each criterion. Results will be shared and agreed, and any disagreements will be addressed through engaging a third reviewer.

For peer review only - http://bmjopen.bmj.com/site/about/guidelines.xhtml

Data synthesis and interpretation

Strategy for data synthesis.

The main outcome in our study is the medicine pricing policy implementation. Policy implementation is typically done within a single country, but where the same policy is implemented in different countries, the analysis will take the specific context of the country into consideration.

In exploring the policy implementation, we will employ established policy theories and frameworks such as Walt and Gilson's policy triangle [14], Baumgartner's punctuated equilibrium [15] and Lipsky's street-level bureaucracy [16], and will also draw upon further theories and frameworks developed or adapted within the reviewed papers.

Where possible, we will compare the effects of the policies in a quantitative synthesis. We anticipate, however, that the heterogeneity of reporting of outcomes and of context may make it impossible to conduct a meta-analysis. In such a situation we will focus on narrative synthesis.

Whilst using qualitative or narrative synthesis approach [17], data related to the medicines pricing policies will be extracted from the Introduction, Methods and possibly Results sections. Data on the effects of policy implementation and the policy implementation approaches, and key influences will be extracted from the Results and Discussion sections. Extracted data will be analysed thematically and will be structured around the specific questions of the review.

The interpretation of the results will follow the identified themes for each review question. For example, in answering the third review question we will divide the factors into facilitators and constraints and potentially will further sub-divide them by their nature (e.g. community issues, health systems issues, wider socio-economic influences).

At the moment, we are not planning analysis of sub-groups or sub-sets. However, depending on the breadth of extracted data we may consider sub-groups such as geographical region (West Africa, East Africa, Southern Africa), setting (urban, rural) or categories of implementers (health facilities, pharmacies).

The cumulative strength of body of evidence will be assessed across the risk of bias and consistency, drawing upon relevant approaches such as GRADE.

Patient and public involvement

No patient involved.

Ethics and dissemination

Ethics approvals are not required for systematic reviews. However, ethics approvals for the wider AMIPS study within which this review is being undertaken have been granted by the ethics committees from the Ghana Health Service (ref GHS-ERC006/02/20) and the University of Leeds School of Medicine (ref MREC 19-060).

We will disseminate results through academic papers and stakeholder workshops in Ghana and other SSA countries where possible. In Ghana, the review results will be complemented by reviews of policy documents. The findings of this review will also be presented at scientific conferences such as the biannual Global Symposia on Health Systems Research and Thematic Working Groups of the Health Systems Global.

For peer review only - http://bmjopen.bmj.com/site/about/guidelines.xhtml

The results of this review will inform empirical investigations of implementation of medicines pricing policies in Ghana through the in-depth interviews and focus groups, and engagements and consultations with policymakers on seeking ways of further improving the implementation of medicines pricing policies in Ghana.

Authors' contributions

AK and TM jointly conceived the study; TM, AK, AC, LB, ADA, TE, IA, JW, IK, NK contributed to the review design and jointly wrote the protocol; TM, AK, AC, LB, ADA, TE, IA, JW, IK, NK read and approved the final version of the manuscript. AK and TM will be the guarantors of the review.

Funding

This research was commissioned by the National Institute for Health Research (NIHR) NIHR Global Health Policy and Systems Research Development Award using UK aid from the UK Government (grant number 130219). The funders did not have any role in developing the protocol and the views expressed in this publication are those of the author(s) and not necessarily those of the NIHR or the Department of Health and Social Care.

Competing interests

None declared

References

1. Bigdeli M, Laing R, Tomson G. Medicines and universal health coverage: challenges and opportunities: BioMed Central, 2015. 1-3
2. Ghebreyesus TA. All roads lead to universal health coverage. *The Lancet Global Health* 2017;5(9):e839-e40.
3. Wirtz VJ, Hogerzeil HV, Gray AL, et al. Essential medicines for universal health coverage. *The Lancet* 2017;389(10067):403-76.
4. World Health O, Regional Office for E. Access to new medicines in Europe : technical review of policy initiatives and opportunities for collaboration and research. 2015
5. World Health Organization. Assessment of medicine pricing and reimbursement systems in health insurance schemes. 2016
6. Bashaar M, Hassali F, ALrasheedy A, et al. Efficacy of international approaches to medicine price regulation and control: A scoping review. *J Appl Pharm Sci* 2017;7(04):227-41.
7. Leopold C, Vogler S, Mantel-Teeuwisse AK, et al. Differences in external price referencing in Europe—a descriptive overview. *Health Policy* 2012;104(1):50-60.
8. Vogler S, Paris V, Ferrario A, et al. How can pricing and reimbursement policies improve affordable access to medicines? Lessons learned from European countries. *Applied health economics and health policy* 2017;15(3):307-21.

For peer review only - http://bmjopen.bmj.com/site/about/guidelines.xhtml

9. Vogler S, Zimmermann N, de Joncheere K. Policy interventions related to medicines: Survey of measures taken in European countries during 2010–2015. *Health Policy* 2016;120(12):1363-77.
10. Nguyen TA, Knight R, Roughead EE, et al. Policy options for pharmaceutical pricing and purchasing: issues for low-and middle-income countries. *Health policy and planning* 2015;30(2):267-80.
11. Moodley R, Suleman F. Evaluating the impact of the single exit price policy on a basket of originator medicines in South Africa from 1999 to 2014 using a time series analysis. *BMC Health Services Research* 2019;19(1):576. doi: 10.1186/s12913-019-4403-8
12. Steyn R, Burger JR, Serfontein JHP, et al. Influence of a new reference-based pricing system in South Africa on the prevalence and cost of antidiabetic medicine: a pilot study. *International Journal of Pharmacy Practice* 2007;15(4):307-11. doi: 10.1211/ijpp.15.4.0009
13. Shamseer L, Moher D, Clarke M, et al. Preferred reporting items for systematic review and meta-analysis protocols (PRISMA-P) 2015: elaboration and explanation. *BMJ : British Medical Journal* 2015;349:g7647. doi: 10.1136/bmj.g7647
14. Walt G, Gilson L. Reforming the health sector in developing countries: the central role of policy analysis. *Health Policy Plan* 1994;9(4):353-70.
15. Baumgartner F, Jones B. Agendas and Instability in American Politics. Chicago, London: University of Chicago Press 1993.
16. Lipsky M. Street-level bureaucracy: dilemmas of the individual in public services New York: Russel Sage Foundation 1980.
17. Snilstveit B, Oliver S, Vojtkova M. Narrative approaches to systematic review and synthesis of evidence for international development policy and practice. *Journal of Development Effectiveness* 2012;4(3):409-29. doi: 10.1080/19439342.2012.710641

For peer review only

For peer review only - http://bmjopen.bmj.com/site/about/guidelines.xhtml

AMIPS project: Flowchart for screening of search results

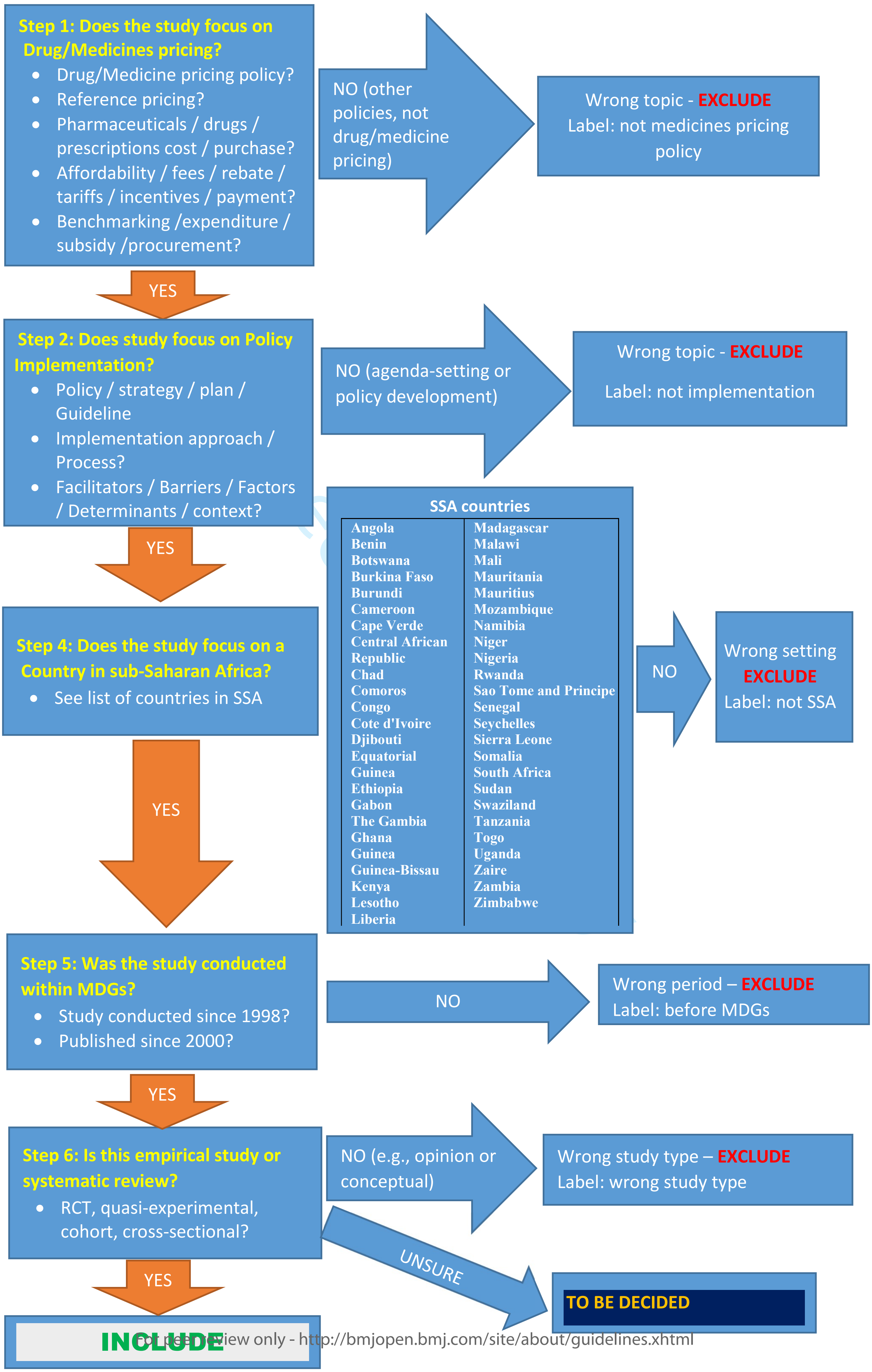

For peer review only - http://bmjopen.bmj.com/site/about/guidelines.xhtml

Along with Medline we intend to search the following databases:

African Index Medicus (via WHO Global Health Index Medicus) all available years; Embase (Ovid) 1996 present; Global Health (Ovid) 1973 to present; Scopus (Elsevier B.V.) 1823 – Present; Web of Science Core Collection: Citation Indexes (Clarivate Analytics) 1900-present;. We will also search for grey literature and French articles in the following; Cairn International (Cairn Info) all available years; Erudit (University of Montreal ???) all available years; IRIS Institutional Repository for Information Sharing (WHO) all available years and World Bank Group Research and Publications

Sample Medline strategy

Ovid MEDLINE(R) and Epub Ahead of Print, In-Process & Other Non-Indexed Citations and Daily <1946 to April 23, 2020>

Search Strategy:

----

1 exp "Africa South of the Sahara"/ (205870)

2 (angola* or benin* or botswana* or "burkina faso" or burundi*).ti,ab,in,kf. (17906)

3 ("cabo verde*" or "cape verde*" or cameroon* or "central africa*" or chad or cormoros or congo* or "ivory coast" or "cote d'ivoire" or djibouti).ti,ab,in,kf. (45277)

4 (guinea* or eritrea* or eswatini* or swaziland* or ethiopia* or gabon* or gambia* or ghana* or guinea*).ti,ab,in,kf. (158364)

5 (kenya* or lesotho* or liberia* or madagasca* or malawi* or mali or mauritania* or mauritius or mozambique*).ti,ab,in,kf. (55553)

6 (namibia* or niger or nigeria* or rwanda*).ti,ab,in,kf. (67659)

7 ("sao tome" or principe* or senegal* or seychelles or "sierra leone*" or somali* or "south africa*" or sudan*).ti,ab,in,kf. (132358)

8 (tanzania* or togo* or uganda* or zambia* or zaire* or zimbabw*).ti,ab,in,kf. (50798)

9 (africa* adj2 ("sub sahara*" or "south* sahara*")).ti,ab,in,kf. (23957)

10 or/1-9 [sub-saharan africa] (519682)

11 Drug Costs/ (15924)

12 ((price? or pricing) adj5 (medicine? or drug? or prescription? or pharmaceutical*)).tw,kw. (4558)

13 ((cost or costs) adj5 (medicine? or drug? or prescription? or pharmaceutical*)).tw,kw. (18930)

14 (afford* adj5 (medicine? or drug? or prescription? or pharmaceutical*)).tw,kw. (1929)

15 (reimburs* adj5 (medicine? or drug? or prescription? or pharmaceutical*)).tw,kw. (2099)

16 (generic* adj5 (medicine? or drug? or prescription? or pharmaceutical*)).tw,kw. (4521)

17 exp fees, pharmaceutical/ (2413)

For peer review only

18 ((purchas* or procur* or expenditure*) adj5 (medicine? or drug? or prescription? or pharmaceutical*)).tw,kw. (4913)

19 ((subsid* or tariff* or incentive* or containment or transparency) adj5 (medicine? or drug? or prescription? or pharmaceutical*)).tw,kw. (2141)

20 ((fee or fees or rebate* or payment* or spend* or saving*) adj5 (medicine? or drug? or prescription? or pharmaceutical*)).tw,kw. (4006)

21 ((benchmark* or cost-plus) adj12 (medicine? or drug? or prescription? or pharmaceutical*)).tw,kw. (641)

22 "low* price* generic*".tw,kw. (76)

23 (essential adj2 (drug* or medicine*)).tw,kw. (3434)

24 Drugs, Essential/ (835)

25 (access* adj3 (medicine? or drug? or prescription? or pharmaceutical*)).tw,kw. (5991)

26 Economics, Pharmaceutical/ (2927)

27 (pharma* adj2 economic*).tw,kw. (833)

28 pharmacoeconomic*.tw,kw. (3898)

29 or/11-28 [drug pricing] (58841)

30 exp policy/ (155119)

31 Government Regulation/ (21073)

32 exp Legislation, Drug/ (32091)

33 ((drug* or medicine* or pharmaceutical* or prescription* or health*) adj7 (guideline* or guidance or policy or policies or law or regulat* or rule* or legislat* or control* or strateg* or framework*)).tw,kw. (538082)

34 (tax or taxes or exemption*).tw,kw. (17307)

35 ((drug* or medicine* or pharmaceutical* or prescription* or health*) adj7 (intervention* or plan* or program*)).tw,kw. (256052)

36 or/30-35 [policy concept -all] (917754)

37 10 and 29 and 36 [SSA and policy] (1190)

38 Health Plan Implementation/ (5829)

39 Program Evaluation/ (62240)

40 (barrier* or facilitator* or challenge* or motivator*).tw,kw. (923252)

41 (implement* or approach* or process*).tw,kw. (3962356)

42 (factor* or determinant* or context*).tw,kw. (3829825)

43 "scal* up".tw,kw. (19228)

For peer review only

44 adopt*.tw,kw. (243593)

45 or/38-44 [implementation] (7698904)

46 10 and 29 and 36 and 45 (763)

47 limit 46 to yr="2000 -Current" (652)

For peer review only

Title: Implementation of medicines pricing policies in sub-Saharan Africa: protocol for a systematic review

PRISMA-P (preferred reporting items for systematic review and meta-analysis protocols) 2015 checklist: recommended items to address in a systematic review protocol

Section and topic	*Item No*	*Checklist item*	*Y/N*	*Page number*	*Line numbers*
**Administrative information**					
Title:					
Identification	1a	Identify the report as a protocol of a systematic review	Y	1	1-2
Update	1b	If the protocol is for an update of a previous systematic review, identify as such	n/a	n/a	n/a
Registration	2	If registered, provide the name of the registry (such as PROSPERO) and registration number	Y	2	67-68
Authors:					
Contact	3a	Provide name, institutional affiliation, e-mail address of all protocol authors; provide physical mailing address of corresponding author	Y	1	6-26
Contributions	3b	Describe contributions of protocol authors and identify the guarantor of the review	Y	9	297-299
Amendments	4	If the protocol represents an amendment of a previously completed or published protocol, identify as such and list changes; otherwise, state plan for documenting important protocol amendments	n/a	n/a	n/a
Support:					
Sources	5a	Indicate sources of financial or other support for the review	Y	9	302-304
Sponsor	5b	Provide name for the review funder and/or sponsor	Y	9	302-304
Role of sponsor or funder	5c	Describe roles of funder(s), sponsor(s), and/or institution(s), if any, in developing the protocol	Y	9	304-306
**Introduction**					
Rationale	6	Describe the rationale for the review in the context of what is already known	Y	4	108-126
Objectives	7	Provide an explicit statement of the question(s) the review will address with reference to participants, interventions, comparators, and outcomes (PICO)	Y	5	129-147
**Methods**					

For peer review only

Section and topic	*Item No*	*Checklist item*	*Y/N*	*Page number*	*Line numbers*
Eligibility criteria	8	Specify the study characteristics (such as PICO, study design, setting, time frame) and report characteristics (such as years considered, language, publication status) to be used as criteria for eligibility for the review	Y	5-6	153-186
Information sources	9	Describe all intended information sources (such as electronic databases, contact with study authors, trial registers or other grey literature sources) with planned dates of coverage	Y	5	154-158
Search strategy	10	Present draft of search strategy to be used for at least one electronic database, including planned limits, such that it could be repeated	Y	6-7	189-212
Study records:					
Data management	11a	Describe the mechanism(s) that will be used to manage records and data throughout the review	Y	7	214-217
Selection process	11b	State the process that will be used for selecting studies (such as two independent reviewers) through each phase of the review (that is, screening, eligibility and inclusion in meta-analysis)	Y	7	219-224
Data collection process	11c	Describe planned method of extracting data from reports (such as piloting forms, done independently, in duplicate), any processes for obtaining and confirming data from investigators	Y	7	226-236
Data items	12	List and define all variables for which data will be sought (such as PICO items, funding sources), any pre-planned data assumptions and simplifications	Y	7-8	228-236
Outcomes and prioritization	13	List and define all outcomes for which data will be sought, including prioritization of main and additional outcomes, with rationale	Y	8	251-273
Risk of bias in individual studies	14	Describe anticipated methods for assessing risk of bias of individual studies, including whether this will be done at the outcome or study level, or both; state how this information will be used in data synthesis	Y	7	237-248
Data synthesis	15a	Describe criteria under which study data will be quantitatively synthesised	n/a	n/a	n/a

For peer review only

Section and topic	*Item No*	*Checklist item*	*Y/N*	*Page number*	*Line numbers*
	15b	If data are appropriate for quantitative synthesis, describe planned summary measures, methods of handling data and methods of combining data from studies, including any planned exploration of consistency (such as I2, Kendall's τ)	n/a	n/a	n/a
	15c	Describe any proposed additional analyses (such as sensitivity or subgroup analyses, meta-regression)	n/a	n/a	n/a
	15d	If quantitative synthesis is not appropriate, describe the type of summary planned	Y	8	261-269
Meta-bias(es)	16	Specify any planned assessment of meta-bias(es) (such as publication bias across studies, selective reporting within studies)	Y	8	258-260
Confidence in cumulative evidence	17	Describe how the strength of the body of evidence will be assessed (such as GRADE)	Y	8	266-275

For peer review only
